# A review of the development and implementation of the critical source area concept: A reflection of Andrew Sharpley's role in improving water quality

**DOI:** 10.1002/jeq2.20551

**Published:** 2024-02-28

**Authors:** Richard McDowell, Peter J. A. Kleinman, Philip Haygarth, Joshua M. McGrath, Douglas Smith, Louise Heathwaite, Antti Iho, Oscar Schoumans, David Nash

**Affiliations:** ^1^ AgResearch, Lincoln Science Centre Lincoln New Zealand; ^2^ Faculty of Agriculture and Life Sciences Lincoln University Lincoln New Zealand; ^3^ USDA ARS Fort Collins Colorado USA; ^4^ Lancaster University Lancaster UK; ^5^ OCP North America Wayzata Minnesota USA; ^6^ USDA Agricultural Research Service Temple Texas USA; ^7^ LUKE, Natural Resources Institute Finland Helsinki Finland; ^8^ Wageningen University and Research Wageningen The Netherlands; ^9^ University of Melbourne Melbourne Victoria Australia

## Abstract

Critical source areas (CSAs) are small areas of a field, farm, or catchment that account for most contaminant loss by having both a high contaminant availability and transport potential. Most work on CSAs has focused on phosphorus (P), largely through the work in the 1990s initiated by Dr. Sharpley and colleagues who recognized the value in targeting mitigation efforts. The CSA concept has been readily grasped by scientists, farmers, and policymakers across the globe. However, experiences and success have been mixed, often caused by the variation in where and how CSAs are defined. For instance, analysis of studies from 1990 to 2023 shows that the proportion of the annual contaminant load coming from a CSA decreases from field to farm to catchment scale. This finding is consistent with increased buffering of CSAs and greater contribution of other sources with scale, or variation in the definition of CSAs. We therefore argue that the best application of CSAs to target mitigation actions should be at small areas that truly account for most contaminant loss. This article sheds light on the development and utilization of CSAs, paying tribute to Dr. Sharpley's remarkable contributions to the improvement of water quality, and reflecting upon where the CSA concept has succeeded or not in reducing contaminant (largely P) loss.

## Core Ideas

- Critical source areas (CSAs) are small areas of a field, farm or catchment that account for most contaminant loss.
- The proportion of the contaminant load coming from a CSA decreases from field to farm to catchment scale.
- The CSA concept was easily understood by scientists, farmers, and policymakers globally.
- Targeting CSAs with mitigation strategies has made those strategies more cost‐effective.
- Dr. Andrew Sharpley was central to the development and implementation of CSAs.

**Abbreviations**
BMPs: best management practices
CSAs: critical source areas

## 1 INTRODUCTION

The concept of critical source areas (CSAs) has changed over time. From our experience and interpretation of how CSAs have developed over time, we define CSAs as “*small areas of a field, farm, or catchment (viz. watershed) that account for most contaminant loss caused by these areas having a high contaminant availability and transport potential*.” This definition is intentionally broad so that it captures multiple contaminants, hydrological flow paths, and spatial and temporal scales. This definition is also easily understood by experts and nonexperts alike and allows stakeholders to easily identify CSAs on their land and have confidence that targeting CSAs with best management practices (BMPs) is a cost‐effective method to mitigate contaminant losses from land to water (Arbab et al., 2018).

**FIGURE 1:**
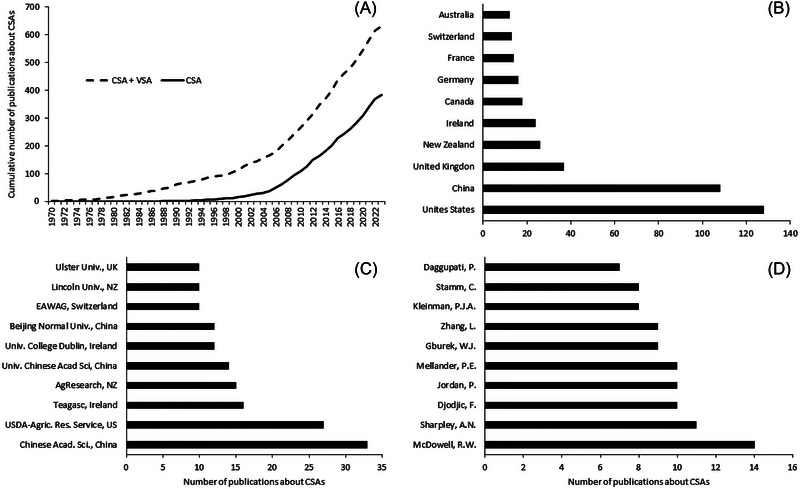
Data showing the cumulative number of publications about critical source areas (CSAs; or variable source area [VSA]) over time (A) and by country (B), institution (C), and contributing author (D). *N* = 383; data were sourced from SCOPUS using the search term “critical source area” in title or abstract.

As a result of the simplicity and applicability of CSAs, different jurisdictions have widely adopted the concept under voluntary schemes and in policy, globally (Figure 1). Their implementation has led to practice change by landowners and associated improvements in water quality (see Section 3). The development of the CSA concept was a collaborative effort involving many contributors (e.g., Figure 1) and advances in science. Dr. Andrew Sharpley was often at the heart of those collaborations and advances. This paper traces the development and implementation of CSAs. We pay particular attention to how the science informed and questioned how contaminants are lost from land to water and use a range of case studies from six countries to demonstrate how the concept has been implemented. Finally, we offer thoughts on where, or if, CSAs can be further developed and implemented to improve water quality.

## 2 DEVELOPMENT

In examining their history, CSAs were first mentioned in the 1970s and 1980s to describe areas and times when and where pesticides and other nonpoint source pollutants were being lost from land to water (Maas et al., 1985). Although still occasionally used to isolate areas of pesticide loss (Doppler et al., 2014), CSAs have also been applied to sediment, the fecal indicator bacterium (*Escherichia coli*), and nitrogen (N), but mainly to phosphorus (P) (Imani et al., 2019; McDowell & Srinivasan, 2009; Oliver et al., 2010; Wei et al., 2017). The attention on P in catchments was a symptom of a need to mitigate P losses—owing to the role of P in controlling eutrophication globally (McDowell, Noble, et al., 2020), but also of the concept's fit to P loss processes, largely focusing on surface runoff (viz. overland flow; Pionke et al., 2000).

As applied to P, the foundation of the CSA concept is that surface runoff, that is, the dominant transport mechanism for P in sloping landscapes (or leaching in flat land), is concentrated to small areas. This aligns with a statistical concept called the Pareto principle, also known as the 80:20 rule (Pareto, 1897). The Pareto principle describes the probability distribution of many societal or physical phenomena such as the size of cities within a country or the ownership of land among landowners. The rule is intuitive, and it has resonated with stakeholders when used as an analogy to justify CSA‐based management.

Indeed, a major motivation for adopting CSA management is to improve the cost‐effectiveness in environmental protection via targeting of resources to areas offering the greatest return on remedial investment. This means achieving given environmental goals with least possible costs or, alternatively, achieving highest possible environmental quality with available resources. When pollution sources and their abatement costs are known, cost‐effective allocation equalizes marginal abatement costs across pollution sources (Baumol & Oates, 1988).

Notably, in using the 80:20 rule to explain CSAs, stakeholders may be left with the impression that this ratio is consistent and constant. As evident from Table 1 and Figure 1, it is not. Variation may be caused by how CSAs are defined and, in its application to P, dynamic processes influencing transport and source properties. When defining a CSA, it is possible to identify a livestock urine patch containing 600–1000 kg N·ha^−1^ (Di & Cameron, 2002) covering about 2% of a grazed grassland field (Lilburne et al., 2012) as a CSA since these patches account for >95% of N losses (Betteridge et al., 2010). However, practically, management of such small patches is problematic, instead resorting to the management of when stock numbers are likely to be on leaky soils at a block scale (with a block being a combination of several fields under similar management) (Beukes et al., 2020). In addition, at larger farm and catchment scales, CSAs tend to account for less contaminant load and cover larger areas as surface runoff processes (dominant at small scales) are diluted by other flow paths and contaminant sources (Table 1; Figure 1). For instance, the dynamic processes of surface runoff flow may expand and contract the areas of runoff that mobilize, or, in the words of Sharpley, activate, P sources in the landscape (Sharpley et al., 2008). Further, changing management and fate of P sources (soils, fertilizers, manures) may impact their spatial distribution, thereby affecting their availability to runoff, over time (Sharpley, 1987). Finally, as scale increases, the diversity of contaminant sources also increases, such as greater contribution of nonagricultural sources, particularly point sources. Therefore, CSA‐informed management of contaminants must balance a variety of factors if it is to be practical and effective (Kleinman, Sharpley, Buda, et al., 2011).

**TABLE 1:** Measurement taken of the relative importance of annual yields of contaminant losses from critical source areas (CSAs) of runoff relative to total losses by land use or management in different jurisdictions.

Scale/contaminant	Years measured	Size (ha)	Land use	Total loss (kg·ha^−1^·year^−1^)	Area considered as CSA (%)	Percent loss accounted for by CSA (%)	Loss from CSAs modeled or observed^a^	Jurisdiction	Reference
Field									
N	2009	0.5	Grazed sheep	–	16	50	Observed	New Zealand	Betteridge et al., 2010
P	1975–1989	4–34	Mixed	0.03–0.5	38	74	Observed	Sweden	Ulén et al., 2001
P, *E.coli*	2006	8	Dairy	2, 1.93^11^ ^e^	5	93, 77	Observed	New Zealand	McDowell, Daly, et al., 2020
P, Sed	1994	9–27	Mixed	–	11, 6	50, 98	Mod/Obs	PA, USA	Pionke et al., 2000
Farm									
N, P	2009–2011	60	Dairy	29, 4.4	15, 15	>50, >50	Mod/Obs	Australia	Adams et al., 2014
P	2008–2010	4.1	Grazed dairy	1.2	1.4	50	Observed	New Zealand	Lucci et al., 2013
P	1993–1995	167	Dairy—CAFO	0.9–7.9	5	37	Mod/Obs	NY, USA	Ghebremichael et al., 2013
P	2004–2007	9–330	Grazed deer, Sheep	0.5–3.0	10–15	80–90	Mod/Obs	New Zealand	McDowell & Srinivasan, 2009
P	2010	824	Grazed dairy	–	10	53	Mod/Obs	Switzerland	Hahn et al., 2013
*E. coli*	2005	147	Grazed grassland	–	5	95	Mod/Obs	France	Trevisan et al., 2010
N, P	2005–2013	673	Mixed	–	7.5, 7.5	61, 55	Modeled	Iran	Imani et al., 2019
P, Sed	1997–2017	265	Arable	100, 0.14	10, 10	75, 73	Observed	Switzerland	Remund et al., 2021
Catchment									
N	2008–2014	622,369	Mixed	–	25^c^	50	Modeled	China	He et al., 2019
N	1990–2010	488,800	Mixed	–	30	30^c^	Modeled	China	Guo et al., 2020
N	2004–2005	256,500	Mixed	–	36	57^c^	Mod/Obs	China	Shang et al., 2012
N	1986–2014	42,348,000	Sugarcane	9.2	1.5%	41%	Modeled	Australia	McCloskey et al., 2021
P	1995–1999	33,500	Irrigated dairy	2	2	20%	Modeled	Australia	Grayson et al., 2001
P	2012–2014	10,800	Grazed sheep	0.8	50	68	Observed	New Zealand	Waters & Webster‐Brown, 2016
P	2011–2012	14,500	Mixed	1.7–5.0	14	30	Mod/Obs	Turkey	Gungor et al., 2016
P	2010	1,792,400	Mixed	–	14	26	Modeled	China	Wen et al., 2021
P	2010–2014	10,890,000	Mixed	–	1.2–23	25^c^	Modeled	China	Lou et al., 2016
P	2005–2009	310,527	Mixed	–	20	74	Mod/Obs	VT, USA	Winchell et al., 2015
P	–	292,000	Mixed	–	40	90	Modeled	China	Li et al., 2016
P	2014–2017	200,000	Annual horticulture	0.5–7.3	1.2	9%	Modeled	Australia	Hall et al., 2018
Sed	1986–2014	42,348,000	Grazing	∼100	71%	53%	Modeled	Australia	McCloskey et al., 2021
Sed	2002–2014	920,000–1,045,000	Mixed	–	26–49	51–74	Modeled	India	Nagireddy et al., 2022
Sed	2011	Albania	Mixed	–	22	93	Modeled	Albania	Kovacs et al., 2012
N, P	2006–2009	64,300	Irrigated dairy	14, 1.8	2, 2	16, 33	Modeled	Australia	Hall, 2011
N, P	2014–2017	200,000	Dryland dairy	3–7, 0.1–1.9	8.5, 8.5	33, 32	Modeled	Australia	Hall et al., 2018
N, P	2007–2017	31,000	Mixed	–	7, 14	53, 76	Mod/Obs	Australia	Lu et al., 2023
N, P	2010–2012	12,300	Mixed	–	12.5, 15.1^b^	22, 33	Modeled	China	Chang et al., 2021
N, P	2004–2012	116,900	Mixed	5.1, 0.4	26, 22	30, 30^c^	Modeled	China	Du et al., 2016
N, P	2010–2018	708,600	Mixed	–	28, 28^c^	49, 45	Modeled	China	Li et al., 2022
N, P	2010	1,792,400	Mixed	4.6, 0.8	16, 13	46, 48	Modeled	China	Zhuang et al., 2016
N, P	2000–2019	Continental US	Mixed	–	8, 2	98, 92	Mod/Obs	United States	Frei et al., 2021
P, Sed	2008–2016	14,300	Mixed	–	6–48, 3–46^d^	20, 20^c^	Modeled	Canada	Shrestha et al., 2021
P, Sed	1973–2008	23,000–197,000	Mixed	–	5, 5	50, 35	Mod/Obs	IA, USA	White et al., 2009
N, P, Sed	2000–2008	57,000	Mixed	–	20, 20, 20^c^	12, 8, 4	Mod/Obs	GA, USA	Niraula et al., 2013

Abbreviation: Sed, sediment.

^a^ Modeled refers to the isolation of CSAs via a modeling approach with or without validation data; Observed refers to in‐field measurements of surface runoff, excretal deposition, leaching, or streamflow from CSAs; Mod/Obs refers to modeled estimated that are validated against observations.

^b^ Area‐weighted mean.

^c^ CSA or loss is user‐defined proportion of total area or load.

^d^ Area varied by season.

^e^ Measured as coliform forming units·100 mL^−1^·ha^−1^.

### 2.1 The arrival of CSAs in understanding and managing P

Major steps in the development of CSAs have focused on our understanding of the source of contaminants and their transport by hydrological processes. Indeed, many early definitions of CSAs, including those of Dr. Sharpley and colleagues, described them as areas where there was a coincidence of a highly available source of P and active hydrology to move P (either in dissolved or particulate form) from land to water either by surface runoff or subsurface flow (also sometimes incorrectly referred to as “leaching,” which describes the dissolution of solutes, but used for simplicity hereon in) (Gburek & Sharpley, 1998).

Dr. Sharpley pioneered the quantification of P availability for surface runoff and leaching, initially with P fractionation techniques in the 1980s that traced the availability of P to plants in response to inorganic and organic P additions (Sharpley, 1987). As a forerunner, Dr. Sharpley pioneered the concept of the “Effective Depth of Interaction” as an early recognition of the selective nature of the interaction between source P and flowing water (Sharpley, 1985). With P enrichment, Dr. Sharpley realized in the 1990s that highly plant‐available P forms were also available to algae in surface water and went on to develop other tests (e.g., Fe‐oxide strips) that were better correlated to algal biomass (Dils & Heathwaite, 1998; Robinson et al., 1994). With colleagues in the 2000s, it was found that the risk of P loss increased rapidly both beyond soil test P concentrations considered optimal for plant growth and sometimes below this optimal level if the soil poorly sorbed P or was disproportionately prone to surface runoff (Buda et al., 2009; McDowell & Sharpley, 2001). These studies have been built upon to isolate where soil chemistry controls the risk of P loss, for example, in soils with a low P sorption capacity and where there is little change in leaching rates (see also the case study in the Netherlands) (Kleinman, Sharpley, McDowell, et al., 2011; McDowell & Monaghan, 2015; Thomas et al., 2016).

**Core Ideas**

- Critical source areas (CSAs) are small areas of a field, farm or catchment that account for most contaminant loss.
- The proportion of the contaminant load coming from a CSA decreases from field to farm to catchment scale.
- The CSA concept was easily understood by scientists, farmers, and policymakers globally.
- Targeting CSAs with mitigation strategies has made those strategies more cost‐effective.
- Dr. Andrew Sharpley was central to the development and implementation of CSAs.

During the 1990s, attention shifted to hydrology as a controlling factor for both the mobilization of P into flow and as a mechanism to transport P to streams (Haygarth & Jarvis, 1999). Data were already beginning to show that without active hydrology, little P would be lost. Indeed, in the late 1990s and early 2000s, the definition of CSAs shifted from merely considering the intersection of source and transport factors, to being dependent on the product (i.e., multiplication) of these factors (Pionke et al., 2000). To describe hydrology, Drs. Bill Gburek and Harry Pionke worked with Dr. Sharpley to incorporate variable source areas (VSAs) (Dunne & Black, 1970). These VSAs in turn describe the generation of “saturation‐excess” surface runoff from near stream areas that would expand or contract in response to rainfall between and during storms (Gburek & Sharpley, 1998; Gburek et al., 2002). This advance enabled CSAs to make a dynamic link to changing soil physical conditions as well as to climate change (Wagena et al., 2018).

From the 2000s, it was becoming clear that transport (hydrology) was likely to be more important in many landscapes than source factors because the scale and range of transport (i.e., range of storms causing surface runoff or leaching) were greater than the range of sources (e.g., P concentrations in most topsoils). This was highlighted in one study in central Pennsylvania (USA) where surface runoff losses and loads (from small plots) at the foot of a slope were approximately seven times those halfway up the slope despite Mehlich‐3 P concentration being enriched in upslope soils relative to soils at the foot of the slope (Buda et al., 2009). Importantly, the concept of “connectivity” emerged in describing CSAs—that is, runoff from agricultural fields must be connected to streams to influence water quality in storm flow (Haygarth et al., 2000; Heathwaite, Quinn, et al., 2005). Additional work showed that when P sorbed to sediment from stormflow settled onto the streambed, P could later dissolve into baseflow and influence algal growth (McDowell, Depree, et al., 2020).

The dominance of hydrology was also clear at the catchment scale, where instream loads were shown to be proportional to storm size and the extent of surface runoff, where sources were stable over space and time (Kleinman et al., 2006; Ockenden et al., 2016; Sharpley et al., 2008). However, variation in P losses was also exacerbated by sources where, for instance, surface runoff quickly followed applications of readily available P such as manure or water‐soluble fertilizers (Nash et al., 2019; Preedy et al., 2001). Clearly, with a complex array of spatial and temporal interactions, successfully mitigating P losses from CSAs required a simple framework that stakeholders could understand—enter the P index.

### 2.2 How implementation of a decision support tool drove CSA development

The P index was developed >30 years ago and aimed to identify parameters that influenced P loss, assess the risk of P loss from field to freshwaters, and help identify BMPs that would mitigate P loss (Lemunyon & Gilbert, 1993). Prior to and for a short time after its development, regulatory authorities were implementing policy based on thresholds of soil test P to limit the likelihood of P loss. However, this resulted in some farmers using the threshold as a target to achieve, often increasing soil test phosphorus in CSAs, while being seen by other farmers and scientists as inflexible and unrepresentative of P loss processes at a catchment scale (Sharpley et al., 2001).

The original P index was underpinned by a combination of fundamental science, as well as field and farmer knowledge. This simplicity and transparency are often cited as key to why the P index was implemented across most of the United States and in several other countries including Denmark, Norway, Sweden, Ireland, and Canada (Andersen & Kronvang, 2006; Bechmann et al., 2005; Hughes et al., 2005; Nelson & Shober, 2012; Reid, 2011; Ulén et al., 2011). As an example of transparency, studies have shown that areas mapped in P indices as CSAs highly align (e.g., correlation coefficient = 0.85) with those considered visually as CSAs by farmers (Djodjic & Markensten, 2019; Djodjic et al., 2018).

Although based on a field‐by‐field evaluation of P loss risk on farm, the location of CSAs often did not coincide with field boundaries. Moreover, normal farm activities within CSAs (e.g., tillage) often meant that areas could become CSAs only temporarily. This variability resulted in many attempts to define CSAs by modeling. These modeling studies have downscaled CSAs with fine‐resolution spatial data (e.g., LIDAR) and combined the results with other hydrologic concepts like the topographic wetness index (Beven & Kirkby, 1979) (or others originating from VSA hydrology), which showed the likelihood of runoff contributing to contaminant losses over time and opened the possibility to forecast losses in the future (Easton et al., 2017). Some have been successful, but often only presented modeled outputs (Babaei et al., 2019; Huang et al., 2021). Others combined modeling results with field observations to yield mixed results, often finding that either the data or models were insensitive to the isolation of CSAs (Heathwaite, Dils, et al., 2005; Page et al., 2005; Reaney et al., 2019).

Many of these studies have incorporated CSAs into models that operate at a field, farm, or catchment scale. However, to be useful, models should always be connected to empirical data and management. Some of the earliest attempts at connecting the modeling of P losses to empirical data and management were achieved by, or under, the supervision of Dr. Sharpley (Sharpley, 1980; Sharpley & Smith, 1989; Sharpley et al., 1982). These studies have been used to drive advice on when and where to apply fertilizer and manure to avoid P loss. Subsequent work on rates and form (often also in collaboration with Dr. Sharpley) have formed the basis of the 4Rs of nutrient management (right rate, right form, right time, and right place).

The identification of CSAs by models or within tools like the P index was always intended to help target BMPs (Lane et al., 2006). Targeting, especially via a map, enables a farmer/landowner to see and concentrate their effort and time on parts of their farm or field. In an analysis of 14 catchments in New Zealand, targeting CSAs was estimated to increase the cost‐effectiveness of BMPs by six to seven times compared to a nontarget approach (McDowell, 2014). Some tools have identified CSAs and linked this to estimates of cost‐effectiveness of BMPs, allowing the user to choose BMPs based on a desired level of P loss reduction and cost (Davison et al., 2008; Gooday et al., 2014; McDowell et al., 2015; Strauss et al., 2007). However, as mentioned previously, the delineation of CSAs remains a problem as it is subjective, dependent upon your spatial scale of interest and how far you wish to deviate from the 80:20 rule. While modeling continues to define CSAs and the cost‐effectiveness of BMPs with ever increasing complexity and precision (Wu et al., 2022), true to the original intent of CSA and the P index, their success in improving water quality will always be tethered to their applicability and practicality for management (i.e., CSAs can be so small that some BMPs may no longer be applicable or practical).

## 3 CASE STUDIES OF IMPACT

**FIGURE 2:**
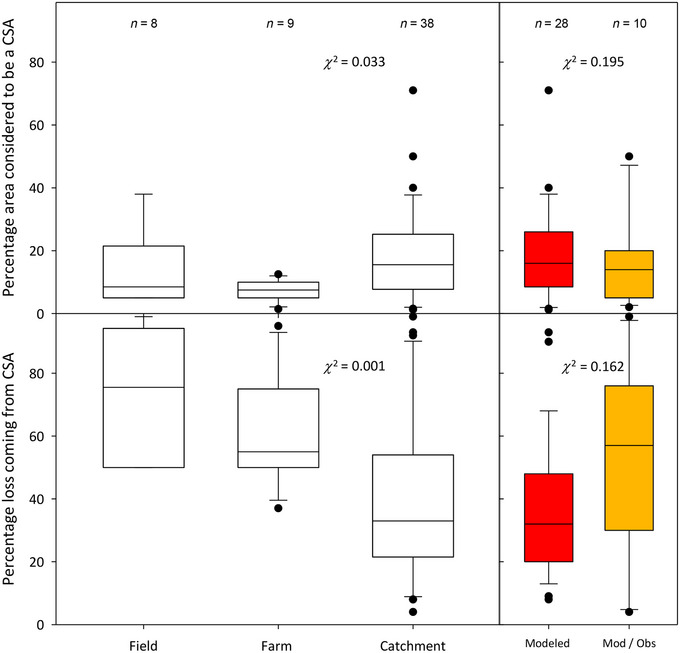
Unfilled box plots showing the median, 25th and 75th percentiles, and the 5th and 95th percentiles (as whiskers) for the percentage area considered to be a critical source area (top) and the percentage loss coming from critical source areas (bottom) for contaminant losses at different scales and filled boxplots using the same criteria for catchment studies whose data were generated by either modeling (only) or a combination of modeling and observations. Also given are the number contaminants (*n* = 8–38), and the output (*χ* ^2^) from a Kruskal–Wallis one‐way analysis of variance contrasting median percentages (for all contaminants pooled together) at each scale and method. Data are from Table 1.

According to a SCOPUS search of the literature, 383 studies have examined CSAs between 1988 and 2023. The authors of these studies come from 39 countries, with most publications from the United States, the United Kingdom, New Zealand, Canada, and Ireland (Figure 2). This reflects not only where the science on CSAs has occurred but, moreover, the tailoring of CSAs for use as a tool to improve water quality in these jurisdictions. Since the CSA concept was design to facilitate impact, we chose a subset of these 39 countries to explore how CSAs have been incorporated into advice or policy and used by farmers to improve water quality.

### 3.1 The United States

The importance of CSAs to P management strategies in the United States can be tied directly to the widespread adoption of the P index (Sharpley et al., 1985, 1979), a tool that Dr. Sharpley helped to develop and implement (Osmond et al., 2023). The adoption of CSA‐based management in the United States emerged from unprecedented collaboration between federal environmental and agricultural agencies, and an ensuing agreement to allow for three approaches to P‐based management on Concentrated Animal Farming Operations (CAFOs) regulated under the Clean Water Act (USDA & USEPA, 1999). Of the three management options promulgated by the CAFO statute (agronomic or environmental soil threshold vs. CSA), the CSA approach, embodied by the P index, was considered most palatable for management and most defensible, scientifically (McDowell et al., 2001; Sharpley et al., 2003). Dr. Sharpley played a critical role in the development of Maryland's P index, the first state policy‐mandated P index in the United States, through Maryland's Mule Barn process, a collaborative process that engaged experts and stakeholders from the greater Chesapeake Bay watershed region (Coale et al., 2002; Osmond et al., 2023).

Phosphorus remains a major cause of eutrophication in agricultural watersheds of the United States, despite widespread implementation of the P index. A key critique of CSA‐based management in the United States has been the extreme variability of the versions of the P index implemented by different states, manifest in variable CSA identification and broad differences in nutrient management recommendations for CSAs from different state P Indices (Osmond, Sharpley, et al., 2012). Similarly, enforcement of P‐based management has been inconsistent (Perez, 2015). Other critiques include the complexity of nutrient management plans, and resource constraints (cost, time) to implementing CSA management required by the P index (Ehmke, 2012). Although curbing eutrophication is profoundly difficult, there have been notable success stories in watersheds where the P index has been implemented, including New York City's drinking water source watersheds in the Catskill Mountains and the Illinois River Watershed of Arkansas and Oklahoma (Osmond, Meals, et al., 2012; Sharpley et al., 2012). More recently, a number of US states have sought to implement dynamic identification of CSAs to allow managers to adjust daily applications based upon current conditions and short‐term forecasts (Easton et al., 2017).

It is remarkable to observe that the empirical foundation of the P index is strongly grounded in settings where Sharpley and select colleagues carried out their science. Relatively few US states have rigorously tested and validated the P index that they have implemented (Butler et al., 2010; Harmel et al., 2005; Veith et al., 2005). Following the guidance of Sharpley et al. (2011), some states have validated P indices against modeled field scale P loss (Bolster et al., 2012). Given the importance of physiographic context and scale to hydrology and associated inferences related to P transport mechanisms, a consistent challenge to the successful implementation of CSA‐based management in the United States has been to accurately represent transport factors, including artificial drainage (tile drains and open ditches) and VSA hydrology, in the P index (King et al., 2015; Kleinman et al., 2015; Shober et al., 2017). In addition, some source factors within the P index have also proven to be problematic to the identification of CSAs, especially the availability of P (viz. legacy P) in watersheds with large P surpluses and freeze/thaw processes that can exacerbate dissolved P release from vegetation (Kleinman, Sharpley, McDowell, et al., 2011; Liu et al., 2018; Sharpley et al., 2013). Long cognizant of these challenges, Dr. Sharpley played an active role in supporting research programs across the United States to improve the P index (Sharpley et al., 2017) and, in later years, became a strong advocate in the United States for addressing barriers faced by farmers and other nutrient managers seeking to balance goals of farm profitability and productivity with goals of environmental betterment (Dodd & Sharpley, 2015). Dr. Sharpley's continued influence on the evolution of the P index can be seen in newer P indexes, like Maryland's Phosphorus Management Tool v2 (PMT2), which elevated the effect of management on overall P transport risk. The PMT2 separated each major P flow pathway into summed components, with each component calculated as the product of source, transport, and management—rather than just source and transport (Fiorellino et al., 2017).

### 3.2 The United Kingdom and Ireland

In the United Kingdom, this work of Dr. Sharpley on CSAs inspired a series of fundamental frameworks and modeling approaches that helped develop new thinking and inform policies for the UK Government. The “phosphorus transfer continuum” was one example, being a conceptual model that separated the P transfer processes into four separate tiers: source, mobilization, delivery, and impact (Haygarth et al., 2005). Sources represented the inputs of P to the system, natural, fertilizer, or animal feed. Mobilization represented the start of P movement from soil and was separated into three subcomponents, solubilization, physical detachment of P from soil, and incidental losses of newly spread sources of P from the soil. Delivery was chosen to represent the hydrological transport from the point of mobilization down the hillslope to the stream (see also the P export and delivery [PEDAL] modeling projects that follow). At the end of the continuum, the impact tier recognized the ecological or economic outcome, which may be a considerable distance and time after the start of P mobilization.

The strength of the transfer continuum approach has been its simplicity. It continues to be well‐cited, owing credit to Dr. Sharpley and co‐workers’ ideas that separated sources from transport factors. This conceptualization has certainly had an impact on educating students and policymakers alike. In the United Kingdom, the continuum approach has contributed to informing policy for assessing cost curves for best P mitigation approaches (Haygarth et al., 2009) and underpinned the wider UK diffuse pollution “User Manual” (Cuttle et al., 2016), which is still used today. The “continuum” has also been applied in Ireland where it has helped their agricultural catchments program formulate new understanding for dairy systems (Murphy et al., 2015) and contributed to framing issues so that Ireland could evaluate its compliance with European Union policies (Wall et al., 2011). More recently, the “continuum” approach has been used to focus the attention on the importance of climate change in relation to the impact of P transfer (Forber et al., 2018).

A family of modeling approaches also owes credit to Dr. Sharpley's work where it aligned with empirical, conceptual, and modeling research at the time. Examples include the work of the PEDAL family of projects (Beven et al., 2005; Brazier et al., 2006; Scholefield et al., 2013; Zhang et al., 2013), the P indicators tool (PIT) (Heathwaite et al., 2003), and the PSYCHIC model (Davison et al., 2008). Much of this research was stimulated by the need to meet head on the challenges set for science by the EU Water Framework Directive. These challenges still exist today. One has only to look at the debacle of the “nutrient neutrality” debate where a requirement to add no more nutrients to rivers prevents additional pollution into rivers by developments. This has halted some housing developments in the border region between England and Wales (Warren, 2023).

### 3.3 New Zealand

There has been a long history of P research in New Zealand ever since early studies by Dr. Sharpley and colleagues in the 1970s who identified hot spots and hot moments of P loss in response to superphosphate applications to near stream areas in wintertime when runoff processes were active (Sharpley & Syers, 1979). Recognition that water quality was declining in response to intensive agriculture leads to a resurgence of P and CSA research from the late 1990s (Gillingham & Thorrold, 2000). Initially, this was focused on providing advice to the fertilizer industry about agronomic and environmental thresholds for P (McDowell, Monaghan, et al., 2003), but then on estimating P loss within an existing model (Overseer) (McDowell et al., 2005). This model was equally owned by the research, government, and fertilizer sectors and was used by fertilizer representatives and ∼50% of farmers. However, as Overseer was not spatial, it was assumed to not capture CSAs despite estimating farm losses reasonably well (*R* ^2^ > 0.85) (Gray et al., 2016).

Work on CSAs in New Zealand has focused on grazed livestock, which present a unique set of behavioral and management characteristics compared to row cropping or CAFOs overseas. These characteristics include year‐round rotational grazing meaning that dung and urine can be applied or deposited in CSAs in winter (McDowell, 2006); treading that can lead to soil disturbance in CSAs (McDowell, Drewry, et al., 2003); the application of dairy shed effluent in early spring when soils are wet (Monaghan & Smith, 2004); and the use of forage crops (to supplement pasture) that are subsequently grazed by large numbers of animals—increasing excretal returns (Burkitt et al., 2017). In addition to the work confirming CSAs associated with VSAs, research in the 2010s also identified CSAs as drinking troughs where livestock camp and laneways that dairy cows use to go to and from the milking shed (Lucci et al., 2013; McDowell, Daly, et al., 2020; Monaghan & Smith, 2012).

Importantly, because of these relatively unique characteristics, New Zealand researchers have created a wide array of BMPs. Most of these BMPs have been costed and tested in multiple regions (McDowell & Nash, 2012). Extension of BMPs has largely relied on farmer‐owned co‐operatives (e.g., fertilizer), industry bodies (e.g., dairy, sheep and beef, deer, arable, horticultural, and viticultural), or regional authorities. This advice has taken the form of information bespoke to a sector, but also tools that extend previous modeling efforts like Overseer to map CSAs and provide estimates of the cost and effectiveness of BMPs suitable to the farm and region (McDowell et al., 2015).

The National Policy Statement for Freshwater Management (NSP‐FM) in 2014 has seen water quality thresholds established, and, since 2020, freshwater farm plans have been made mandatory for all farmers who, depending on the production system, own land >5–20 ha (Ministry for the Environment, 2022). Importantly, CSAs are mentioned in the NPS‐FM as an underpinning concept within freshwater farm plans, and in at least 77 industry and regional sector advice or policy documents. They are also recognized by New Zealand farmers (McDowell et al., 2019).

Beyond raising recognition, the introduction of CSAs and CSA management has been implicated as a major cause for why nationally the proportion of total phosphorus concentrations at sites dominated by intensively grazed pasture has improved (41% showing improving trends between 1994 and 2013, increasing to 65% between 2004 and 2018) (McDowell et al., 2019). Recently, a more direct link has been made between actions to reduce P loss in multiple farms over a 20‐year period in five dairy‐dominated catchments ranging in size from 598 to 2480 ha. The decreases in P losses were attributed to targeting CSAs with BMPs like limiting effluent applications, removing winter forage crops, or shifting from flood to spray irrigation (McDowell et al., 2023).

### 3.4 Australia

**FIGURE 3:**
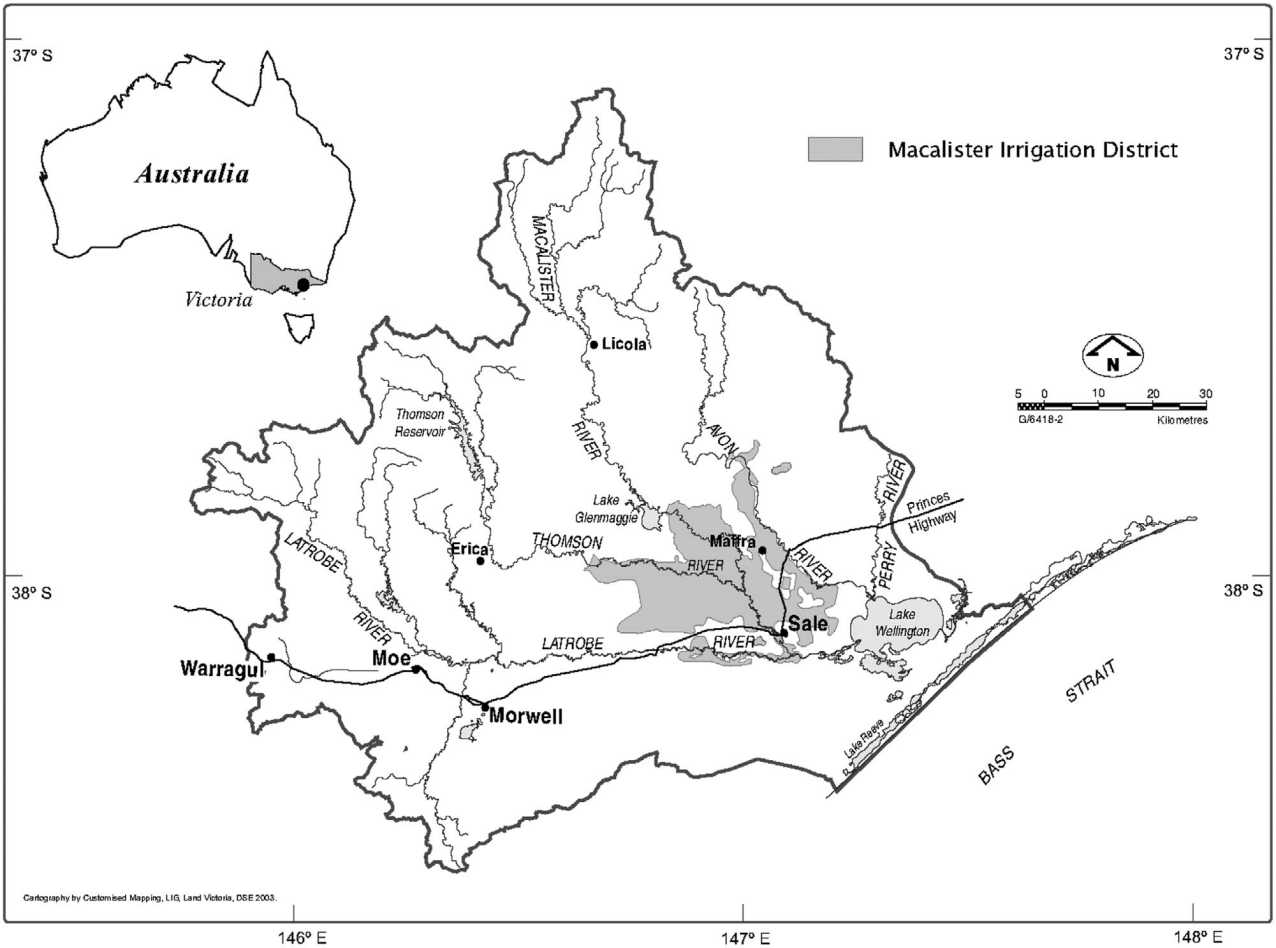
The Lake Wellington catchment in south‐eastern Australia, with the Macalister Irrigation District highlighted.

Water quality issues, including toxic algal blooms, have been an enduring and expensive problem in Australia (Verhoeven, 1992) where CSAs have generally been identified through modeling exercises (Grayson et al., 2001; Hall, 2011; Hall et al., 2018; McCloskey et al., 2021). One good example of where the concept of a CSA has been successfully implemented is the Gippsland Lakes (Figure 3), an internationally significant wetland (Australian Site Number 21, Ramsar Convention on Wetlands of International Importance Especially as Waterfowl Habitat). Most P enters the Gippsland Lakes from the western catchments into Lake Wellington (Grayson et al., 2001). Of P sources, irrigated agriculture in the Macalister Irrigation District (MID) is prominent. The MID comprises <2% (33,500 ha) of the land area and 20% of the P exports to the Gippsland Lakes (Ladson & Tilleard, 2006). In the late 1990s, the MID was identified as being “critical” to the health of the Gippsland Lakes, and a 40% reduction in the 70 t TP·year^−1^ load was mandated (Victorian Environment Protection Authority, 1995).

To achieve the 40% reduction, a joint research/demonstration/extension project titled the Action on Nutrients for Sustainable Agriculture (ANSA) was initiated. This award‐winning project was built on the scientific foundation provided by Dr. Andrew Sharpley and received industry, community, and government support. The extension messages were simple but effective. For example, advice such as “When you try to wash fertilizer in, you wash it off” advised farmers that, as well as adversely affecting downstream water resources, fertilizer application immediately before rainfall or irrigation is both unnecessary and wasteful. In addition to land managers, influencers in the form of farmer “champions” and service providers (e.g., fertilizer spreaders) were also targeted. Research data from a community research farm and commercial farms were used to support the extension messages. Given the initial reluctance of the farming community to engage with the government on water quality issues, all activities emphasized high entertainment values and nonthreatening social interactions. For example, one activity promoting the appropriate management of dairy shed effluent involved a morning bus ride to a local Air Force base to look over fighter jets, a farm visit, a and barbeque lunch. Participants then went home to milk and returned that evening for a bush dance and floor show. Such innovative approaches were used to address both source (i.e., incidental) and transport (e.g., minimum outwash, tailwater reuse) factors in an environment of limited regulation. Ultimately, the ANSA project morphed into more conventional, independent research and extension activities.

Targeting the MID as a CSA has been successful with the 42 t TP·year^−1^ target being met in 10 out of 16 years since 2000 (WGCMA, 2018), and there is a clear trend of continued decreasing P loads into the Gippsland Lakes (EGCMA, 2021). An additional 7.5 t TP·year^−1^ (i.e., 18%) reduction in average annual P loads from irrigation sources in the Lake Wellington catchment by 2030 has now been mandated (Victorian Environment Protection Authority, 2018). The scientific and personal contributions of Dr. Sharpley have been seminal to nutrient export mitigation in Gippsland.

### 3.5 Finland

Agriculture in Southwest Finland is the last pollution hot spot in the Nordic countries, as indicated by the Helsinki Commission, which governs the protection of the Baltic Sea (https://helcom.fi/action‐areas/industrial‐municipal‐releases/helcom‐hot‐spots/). Anthropogenic P loading from the Baltic Sea's watershed is 84% (Iho et al., 2023). Southwest Finland is also the country's most productive agricultural region. For decades, P applications have exceeded the crop uptake, gradually elevating the soil test P values in the region (Uusitalo et al., 2007). Intensive animal production is one of the drivers of P accumulation (Sharpley et al., 2001).

The CSA concept brings scientific precision into real‐world P management, but in Finland, few examples of CSA exist. One reason could be the extensive agri‐environmental policy. Since the EU membership in 1995, the basic measures in Finland's agri‐environmental scheme under the EU Common Agricultural Policy (CAP) have been designed to be as expansive as possible (Lankoski, 2006). In 2015, about 86% of farmers (90% of farmland) were beneficiaries of the scheme. The national agri‐environmental schemes compensate a proportion of the costs of environmental measures for farmers. Together with the Czech Republic, Finland has been the only member state to compensate 100% of the costs. Furthermore, Finland is the only member state adding a full transaction cost of 20% to the compensation (Keenleyside et al., 2011). Hence, the agri‐environmental scheme plays a role as an income support. For a farm incurring lower than average costs for the environmental measures, the income support may be substantial. The Finnish agri‐environmental policy aims at extensive coverage, not at spatial precision. This contradicts the principles of CSA.

Nevertheless, some policies have a clear CSA flavor. First, soil test P thresholds are used to limit P applications in fields that are P enriched and likely to produce runoff. Previously, the limits were a part of the agri‐environmental program, but in 2023, they were made legally binding for all (Government Decree 64/2003). The idea is to bring down the soil test P values particularly in animal farming regions where the participation rate in the agri‐environmental scheme has been lower. The environmental and economic gains of basing P application more precisely on soil test P values are substantial (Iho & Laukkanen, 2012).

Second, land‐use planning carried out in 13 regional governmental centers has designed the spatial allocation of buffer zones on agricultural fields most prone to erosion (Alahuhta et al., 2010). The wide buffer zones are effective in mitigating P loading in surface runoff, particularly from conventionally tilled fields (Uusi‐Kämppä & Jauhiainen, 2010). Unfortunately, the CSA principle does not extend to incentives: the hectare‐based subsidy for establishing buffer zones is identical for all farms. This exemplifies the trade‐off between extensive coverage and precision.

Third, certain projects and pilots operating outside the CAP have put forth CSA principles in practice. For instance, a reverse auction pilot conducted in Southern Finland in 2010 had farmers suggesting a parcel‐specific compensation for applying gypsum (Iho et al., 2014). The bids were ranked according to a compensation P index ratio. The P index was determined by the soil test P, the erodibility estimate, and the proximity to different types of surface waters. Essentially, the approach combined the heterogeneity in P loading risk with the heterogeneity in costs of the measure (expressed in the non‐coordinated bids of the farmers). The pilot showed a substantial efficiency potential in applying CSA principles, compared to the existing, extensive one‐size‐fits‐all management. The current strategic program of the Finnish Government commits to further develop and trial similar mechanisms to improve the spatial precision and the cost‐effectiveness of agri‐environmental policies in Finland (Finnish Government, 2023).

### 3.6 The Netherlands

The Netherlands has a long history of identifying agricultural lands that pose a risk of P polluting groundwater and surface water (Schoumans, 2015). Due to the strong emergence of intensive livestock farming in the mid‐1970s, the vulnerability of different soil types in terms of P binding was mapped (Schoumans, 2015). Since the Netherlands is a flat country with shallow groundwater levels (<40 cm below surface), most attention was paid to P leaching through the topsoil and upper groundwater to local surface waters (trenches, ditches, brooks, etc.). Variation in leaching rates caused by changes in soil types can be used to define those soils that leach the most P as CSAs. An indicator for acid sandy soils was developed based on total P accumulation and the P binding capacity of the soil to show the risk of increased groundwater P concentrations (van der Zee & van Riemsdijk, 1988). This indicator was called phosphate saturation degree, and CSA maps of phosphate saturation degree for the Netherlands were made based on stratified soil sampling protocols for soils types (Schoumans & Chardon, 2015).

**FIGURE 4:**
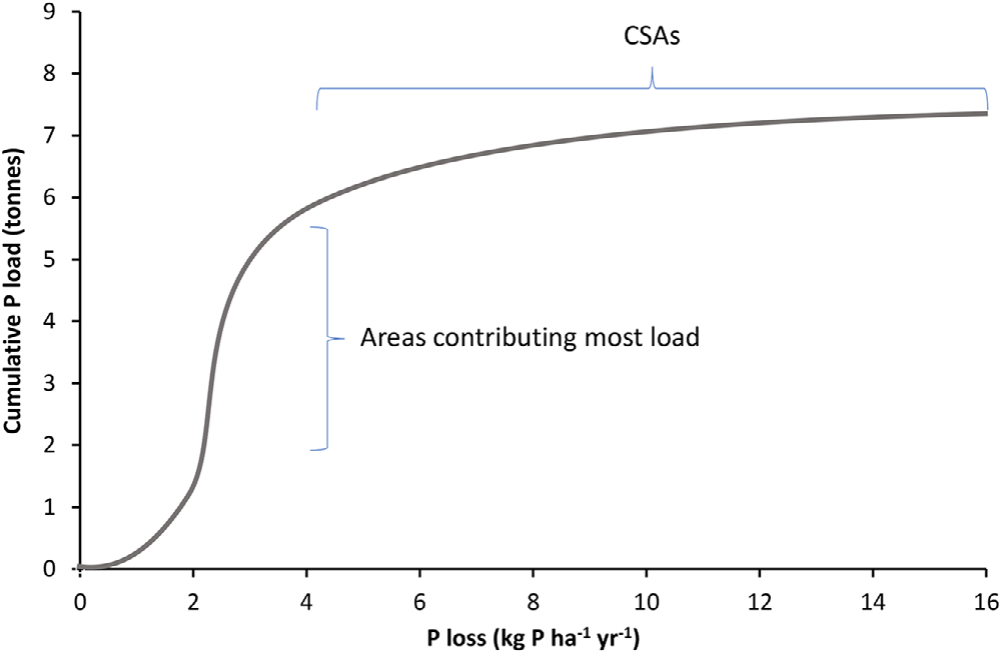
Cumulative distribution of fields contributing to the P load discharging from the Schuitenbeek catchment in the Netherlands.

However, to implement remedial measures in the right place, information about real P losses was needed instead of the potential risk. Furthermore, a more direct link with agronomic parameters (soil test phosphorus) was requested to bridge the gap between environmental impact and the agronomic P management of a field. Based on a description of P sorption in soils and hydrological processes, a simplified P leaching tool, PLEASE, was developed, which predicted P load from a field to nearby surface water (Schoumans et al., 2013). The field measurements required were soil P test (water extractable P), oxalate extractable Al and Fe, information about groundwater fluctuation, and the distance to trenches and ditches (Schoumans et al., 2013). In the Schuitenbeek catchment, where these data were collected, the cumulative distribution of the P load of the surface water showed that some of the fields were hot spots of P loss to surface waters (4–14 kg P·ha^−1^; Figure 4) and perhaps may have been defined as CSAs. However, at a catchment scale, these high‐loss fields represented 4% of the area and accounted for 10% of the load, whereas fields losing a moderate amount of P (about 2–4 kg P·ha^−1^) accounted for about 60% of the load (Figure 4) (Schoumans & Chardon, 2003). This example clearly shows that defining CSAs as the highest P loss fields on a per hectare basis would be an inefficient method of targeting mitigation strategies and reducing P loss.

## 4 FUTURE THOUGHTS

There are some instances where the management of CSAs may fail to improve water quality. These are caused by spatial and temporal variability that influence how a CSA is defined and managed. An example here is where CSAs are unclear and hence unlikely to account for most contaminant losses. This may arise where there is a lack of slope inhibiting VSA hydrology, or where there is no contrast in the leaching potential (or sorption capacity) of soil types. Another example occurs under high rainfall where CSAs are overwhelmed by runoff across all the catchment (Lane et al., 2006; Ockenden et al., 2017). With the likelihood of high‐intensity storms becoming more frequent in many parts of the globe (Wagena et al., 2018), we may see the benefit of their management decline, that is, non‐CSAs are likely to become more important sources of catchment P loss. Alternatively, the location of CSAs may change as crop and livestock production systems adapt to increasing pressure from climatic variation and the need for more food (Bock et al., 2018; Mogollón et al., 2021). However, even where CSAs are well defined, and, for example, contribute 80% of contaminant loss, if the catchment is nutrient rich, then in mitigating this loss, the remaining 20% may still breach water quality thresholds: the “legacy P” conundrum, another concept advanced by Dr. Sharpley (Sharpley et al., 2013).

Moving beyond the definition of CSAs, their management is also impaired because CSAs are often subjectively defined across a spatial and temporal continuum; hence, while a CSA may be spatially defined at a field scale, at a catchment scale the same field may not be a CSA. An obvious example is rill erosion and P loss from parts of a field that are inconsequential in catchment loads dominated by bank erosion (Hayes et al., 2023; Margenot et al., 2023). In addition, CSAs are often active temporarily and may contribute contaminants at a time that is, for example, seasonally insignificant or inconsequential compared to legacy P inputs from soils and sediments. An example is the loss of P from CSAs during winter that is swept downstream and therefore unlikely to cause periphyton growth locally in summer, or inputs during summer that fail to influence periphyton growth because sediment has been deposited locally in winter from a site upstream with a legacy of eroding low‐P‐ and high‐P‐sorbing soil.

As a management tool, CSAs do reinforce and promote cost‐effective mitigation of diffuse pollution. How the CSAs of a region are eventually determined should reflect their net benefits. There are costs to being temporally and spatially more precise in tracking pollution. These should be outweighed by the benefits in terms of lower environmental pressure or management costs. The Finnish and the Netherlands case studies illustrate that broad coverage of existing policies may hinder targeting spatially differentiated measures in practice. Their systems can handle differentiated fertilization limits based upon soil test P, but, without more spatially refined data, more precisely planned measures could not be implemented. These case studies also convey a general lesson: were the agri‐environmental policies results based, it would be in everyone's interest to develop and utilize tools that support precise allocation of environmental management efforts.

In reiterating the intent of Dr. Sharpley, the purpose of CSAs was to provide a framework to focus management efforts on improving water quality outcomes, consistent with observations from select watershed related to the diffuse, agricultural sources of P. While there is merit in continuing to develop the science behind what is a CSA, this must always be linked to what is practical to do by the farmer. For instance, while the technology may exist to manage disjunct, small areas of a field with bespoke mitigations, intuitively this would be difficult to convince a farmer to do manually. In contrast, some potential exists under automated systems, more common in flat land that is easy to mechanize or modify existing infrastructure. Already we see large‐scale uniform‐rate irrigation systems being changed to variable‐rate irrigation systems that account for the water‐holding potential of different soil types (reducing leaching) but are also able to apply fertilizers through irrigation water so that small areas (e.g., 5 m^2^) only have the nutrient they need (McDowell, 2017). Some progress has been made on sloping land with the use of variable‐rate topdressing either by plane or by drone (White et al., 2017), but mitigations outside of changing the 4Rs of fertilizer practice on sloping land are rare.

In conclusion, work over the last 30 years has revealed the significant potential to mitigate contaminant losses from land to water. Because CSAs are easy to explain and, in many landscapes, easy to define, they have also been implemented resulting in material water quality improvement. With increasing pressure on our water resources from primary production and expectation from the public that our waters are clean, there is a clear role for expanding the implementation of CSAs beyond the case studies outlined in this review. However, we also recognize that managing CSAs at some scales may not be appropriate. The modeled and observational data summarized here, and our cases studies, suggest that defining CSAs is simplest and costs lowest at the field scale, where CSAs are most obvious, but considerably more difficult at the catchment scale. In addition, mitigating contaminant losses from CSAs may not be enough to reach water quality objectives. In this scenario, deeper change may be required. In some cases, this may mean the complete removal of nutrient inputs from one catchment to another (e.g., manure) (Spiegal et al., 2020), but land‐use change in others (McDowell et al., 2021). Whichever option is taken, it is inevitable that new CSAs will arise, securing the legacy of Dr. Sharpley and those who worked with him to develop and champion the concept.

## AUTHOR CONTRIBUTIONS

**Richard McDowell**: Conceptualization; data curation; formal analysis; investigation; methodology; project administration; writing—original draft. **Peter J. A. Kleinman**: Conceptualization; writing—original draft; writing—review and editing. **Philip Haygarth**: Writing—original draft; writing—review and editing. **Joshua M. McGrath**: Writing—review and editing. **Douglas Smith**: Writing—review and editing. **Louise Heathwaite**: Writing—review and editing. **Antti Iho**: Writing—original draft; writing—review and editing. **Oscar Schoumans**: Writing—original draft; writing—review and editing. **David Nash**: Writing—original draft; writing—review and editing.

## CONFLICT OF INTEREST STATEMENT

The authors declare no conflicts of interest.

## References

[jeq220551-bib-0001] Adams, R. , Arafat, Y. , Eate, V. , Grace, M. R. , Saffarpour, Sh. , Weatherley, A. J. , & Western, A. W. (2014). A catchment study of sources and sinks of nutrients and sediments in south‐east Australia. Journal of Hydrology, 515, 166–179. 10.1016/j.jhydrol.2014.04.034

[jeq220551-bib-0002] Alahuhta, J. , Hokka, V. , Saarikoski, H. , & Hellsten, S. (2010). Practical integration of river basin and land use planning: Lessons learned from two Finnish case studies. The Geographical Journal, 176(4), 319–333. 10.1111/j.1475-4959.2010.00365.x

[jeq220551-bib-0003] Andersen, H. E. , & Kronvang, B. (2006). Modifying and evaluating a P index for Denmark. Water, Air, and Soil Pollution, 174(1–4), 341–353. 10.1007/s11270-006-9123-0

[jeq220551-bib-0004] Arbab, N. N. , Collins, A. R. , & Conley, J. F. (2018). Projections of watershed pollutant loads using a spatially explicit, agent‐based land use conversion model: A case study of Berkeley County, West Virginia. Applied Spatial Analysis and Policy, 11(1), 147–181. 10.1007/s12061-016-9197-z

[jeq220551-bib-0005] Babaei, H. , Nazari‐Sharabian, M. , Karakouzian, M. , & Ahmad, S. (2019). Identification of critical source areas (CSAs) and evaluation of best management practices (BMPs) in controlling eutrophication in the Dez River basin. Environments, 6(2), 20. 10.3390/environments6020020

[jeq220551-bib-0006] Baumol, W. J. , & Oates, W. E. (1988). The theory of environmental policy (2nd ed.). Cambridge University Press.

[jeq220551-bib-0007] Bechmann, M. , Krogstad, T. , & Sharpley, A. (2005). A phosphorus index for Norway. Acta Agriculturae Scandinavica, Section B—Soil & Plant Science, 55(3), 205–213. 10.1080/09064710510029088

[jeq220551-bib-0008] Betteridge, K. , Costall, D. , Balladur, S. , Upsdell, M. , & Umemura, K. (2010). Urine distribution and grazing behaviour of female sheep and cattle grazing a steep New Zealand hill pasture. Animal Production Science, 50(6), 624–629. 10.1071/AN09201

[jeq220551-bib-0009] Beukes, P. C. , Gregorini, P. , Cameron, K. , & Attwood, G. T. (2020). Farm‐scale carbon and nitrogen fluxes in pastoral dairy production systems using different nitrogen fertilizer regimes. Nutrient Cycling in Agroecosystems, 117(1), 1–12. 10.1007/s10705-020-10052-2

[jeq220551-bib-0010] Beven, K. , Heathwaite, L. , Haygarth, P. , Walling, D. , Brazier, R. , & Withers, P. (2005). On the concept of delivery of sediment and nutrients to stream channels. Hydrological Processes, 19(2), 551–556. 10.1002/hyp.5796

[jeq220551-bib-0011] Beven, K. J. , & Kirkby, M. J. (1979). A physically based, variable contributing area model of basin hydrology /Un modèle à base physique de zone d'appel variable de l'hydrologie du bassin versant. Hydrological Sciences Bulletin, 24(1), 43–69. 10.1080/02626667909491834

[jeq220551-bib-0012] Bock, M. , Gasser, P.‐Y. , Pettapiece, W. W. , Brierley, A. J. , Bootsma, A. , Schut, P. , Neilsen, D. , & Smith, C. A. S. (2018). The land suitability rating system is a spatial planning tool to assess crop suitability in Canada. Frontiers in Environmental Science, 6, 77. 10.3389/fenvs.2018.00077

[jeq220551-bib-0013] Bolster, C. H. , Vadas, P. A. , Sharpley, A. N. , & Lory, J. A. (2012). Using a phosphorus loss model to evaluate and improve phosphorus indices. Journal of Environmental Quality, 41(6), 1758–1766. https://www.agronomy.org/publications/jeq/abstracts/41/6/1758 23128733 10.2134/jeq2011.0457

[jeq220551-bib-0014] Brazier, R. , Schärer, M. , Heathwaite, L. , Beven, K. , Scholefield, P. , Haygarth, P. , Hodgkinson, R. , Walling, D. , & Withers, P. (2006). A framework for predicting delivery of phosphorus from agricultural land using a decision‐tree approach. In J. S. Rowan , A. Werritty , & R. W. Duck (Eds.), Sediment dynamics and the hydromorphology of fluvial systems (pp. 514–523). IAHS.

[jeq220551-bib-0015] Buda, A. R. , Kleinman, P. J. A. , Srinivasan, M. S. , Bryant, R. B. , & Feyereisen, G. W. (2009). Effects of hydrology and field management on phosphorus transport in surface runoff. Journal of Environmental Quality, 38(6), 2273–2284. 10.2134/jeq2008.0501 19875784

[jeq220551-bib-0016] Burkitt, L. L. , Winters, J. L. , & Horne, D. J. (2017). Sediment and nutrient losses under winter cropping on two Manawatu hill country soils. Proceedings of the New Zealand Grassland Association, 79, 19–26.

[jeq220551-bib-0017] Butler, D. M. , Franklin, D. H. , Cabrera, M. L. , Risse, L. M. , Radcliffe, D. E. , West, L. T. , & Gaskin, J. W. (2010). Assessment of the Georgia Phosphorus Index on farm at the field scale for grassland management. Journal of Soil and Water Conservation, 65(3), 200–210. 10.2489/jswc.65.3.200

[jeq220551-bib-0018] Chang, D. , Lai, Z. , Li, S. , Li, D. , & Zhou, J. (2021). Critical source areas’ identification for non‐point source pollution related to nitrogen and phosphorus in an agricultural watershed based on SWAT model. Environmental Science and Pollution Research, 28(34), 47162–47181. 10.1007/s11356-021-13973-9 33886049

[jeq220551-bib-0019] Coale, F. J. , Sims, J. T. , & Leytem, A. B. (2002). Accelerated deployment of an agricultural nutrient management tool. Journal of Environmental Quality, 31(5), 1471–1476. 10.2134/jeq2002.1471 12371163

[jeq220551-bib-0020] Cuttle, S. P. , Newell‐Price, J. P. , Harris, D. , Chadwick, D. R. , Shepherd, M. A. , Anthony, S. G. A. , Macleod, C. J. A. , Haygarth, P. M. , & Chambers, B. J. (2016). A method‐centric ‘User Manual’ for the mitigation of diffuse water pollution from agriculture. Soil Use and Management, 32(S1), 162–171. 10.1111/sum.12242

[jeq220551-bib-0021] Davison, P. S. , Withers, P. J. A. , Lord, E. I. , Betson, M. J. , & Strömqvist, J. (2008). PSYCHIC ‐ A process‐based model of phosphorus and sediment mobilisation and delivery within agricultural catchments. Part 1: Model description and parameterisation. Journal of Hydrology, 350(3–4), 290–302. 10.1016/j.jhydrol.2007.10.036

[jeq220551-bib-0022] Di, H. J. , & Cameron, K. C. (2002). Nitrate leaching in temperate agroecosystems: Sources, factors and mitigating strategies. Nutrient Cycling in Agroecosystems, 46, 237–256.

[jeq220551-bib-0023] Dils, R. M. , & Heathwaite, A. L. (1998). Development of an iron oxide‐impregnated paper strip technique for the determination of bioavailable phosphorus in runoff. Water Research, 32(5), 1429–1436. 10.1016/S0043-1354(97)00346-1

[jeq220551-bib-0024] Djodjic, F. , Elmquist, H. , & Collentine, D. (2018). Targeting critical source areas for phosphorus losses: Evaluation with soil testing, farmers’ assessment and modelling. Ambio, 47(1), 45–56. 10.1007/s13280-017-0935-5 28779474 PMC5709264

[jeq220551-bib-0025] Djodjic, F. , & Markensten, H. (2019). From single fields to river basins: Identification of critical source areas for erosion and phosphorus losses at high resolution. Ambio, 48(10), 1129–1142. 10.1007/s13280-018-1134-8 30569436 PMC6722166

[jeq220551-bib-0026] Dodd, R. J. , & Sharpley, A. N. (2015). Recognizing the role of soil organic phosphorus in soil fertility and water quality. Resources, Conservation and Recycling, 105, 282–293. 10.1016/j.resconrec.2015.10.001

[jeq220551-bib-0027] Doppler, T. , Lück, A. , Camenzuli, L. , Krauss, M. , & Stamm, C. (2014). Critical source areas for herbicides can change location depending on rain events. Agriculture, Ecosystems and Environment, 192, 85–94. 10.1016/j.agee.2014.04.003

[jeq220551-bib-0028] Du, X. , Su, J. , Li, X. , & Zhang, W. (2016). Modeling and evaluating of non‐point source pollution in a semi‐arid watershed: Implications for watershed management. Clean ‐ Soil, Air, Water, 44(3), 247–255. 10.1002/clen.201400773

[jeq220551-bib-0029] Dunne, T. , & Black, R. D. (1970). Partial area contributions to storm runoff in a small New England watershed. Water Resources Research, 6(5), 1296–1311. 10.1029/WR006i005p01296

[jeq220551-bib-0030] East Gippsland Catchment Management Authority (EGCMA) . (2021). Gippsland Lakes Environment Report 2021 . EGCMA.

[jeq220551-bib-0031] Easton, Z. M. , Kleinman, P. J. A. , Buda, A. R. , Goering, D. , Emberston, N. , Reed, S. , Drohan, P. J. , Walter, M. T. , Guinan, P. , Lory, J. A. , Sommerlot, A. R. , & Sharpley, A. (2017). Short‐term forecasting tools for agricultural nutrient management. Journal of Environmental Quality, 46(6), 1257–1269. 10.2134/jeq2016.09.0377 29293860

[jeq220551-bib-0032] Ehmke, T. (2012). The 4Rs of nutrient management. Crops & Soils, 45(5), 4–10.

[jeq220551-bib-0033] Finnish Government . (2023). A strong and committed Finland: Programme of Prime Minister Petteri Orpo's Government 20 June 2023 . Author.

[jeq220551-bib-0034] Fiorellino, N. M. , Mcgrath, J. M. , Vadas, P. A. , Bolster, C. H. , & Coale, F. J. (2017). Use of Annual Phosphorus Loss Estimator (APLE) model to evaluate a phosphorus index. Journal of Environmental Quality, 46(6), 1380–1387. 10.2134/jeq2016.05.0203 29293844

[jeq220551-bib-0035] Forber, K. J. , Withers, P. J. A. , Ockenden, M. C. , & Haygarth, P. M. (2018). The phosphorus transfer continuum: A framework for exploring effects of climate change. Agricultural & Environmental Letters, 3(1), 180036. 10.2134/ael2018.06.0036

[jeq220551-bib-0036] Frei, R. J. , Lawson, G. M. , Norris, A. J. , Cano, G. , Vargas, M. C. , Kujanpää, E. , Hopkins, A. , Brown, B. , Sabo, R. , Brahney, J. , & Abbott, B. W. (2021). Limited progress in nutrient pollution in the U. S. caused by spatially persistent nutrient sources. PLoS ONE, 16(11 November), e0258952. 10.1371/journal.pone.0258952 34843503 PMC8629290

[jeq220551-bib-0037] Gburek, W. , Drungil, C. , Srinivasan, M. , Needelman, B. , & Woodward, D. (2002). Variable‐source‐area controls on phosphorus transport: Bridging the gap between research and design. Journal of Soil & Water Conservation, 57(6), 534–543.

[jeq220551-bib-0038] Gburek, W. J. , & Sharpley, A. N. (1998). Hydrologic controls on phosphorus loss from upland agricultural watersheds. Journal of Environmental Quality, 27(2), 267–277.

[jeq220551-bib-0039] Ghebremichael, L. T. , Veith, T. L. , & Hamlett, J. M. (2013). Integrated watershed‐ and farm‐scale modeling framework for targeting critical source areas while maintaining farm economic viability. Journal of Environmental Management, 114, 381–394. 10.1016/j.jenvman.2012.10.034 23195139

[jeq220551-bib-0040] Gillingham, A. G. , & Thorrold, B. S. (2000). A review of New Zealand research measuring phosphorus in runoff from pasture. Journal of Environmental Quality, 29, 88–96.

[jeq220551-bib-0041] Gooday, R. D. , Anthony, S. G. , Chadwick, D. R. , Newell‐Price, P. , Harris, D. , Duethmann, D. , Fish, R. , Collins, A. L. , & Winter, M. (2014). Modelling the cost‐effectiveness of mitigation methods for multiple pollutants at farm scale. Science of the Total Environment, 468–469, 1198–1209. 10.1016/j.scitotenv.2013.04.078 23706481

[jeq220551-bib-0042] Gray, C. W. , Wheeler, D. M. , McDowell, R. , & Watkins, N. L. (2016). Overseer and phosphorus: Strengths and weaknesses. In L. D. Currie & R. Singh (Eds.), Integrated nutrient and water management for sustainable farming (pp. 1–32). Fertilizer and Lime Research Centre. https://flrc.massey.ac.nz/publications.html

[jeq220551-bib-0043] Grayson, R. , Tan, K. , & Western, A. (2001). Estimation of sediment and nutrient loads into the Gippsland Lakes. CSIRO.

[jeq220551-bib-0044] Gungor, K. , Karakaya, N. , Evrendilek, F. , Akgul, S. , Baskan, O. , Cebel, H. , Farhoud, H. J. , Turkecan, O. , Yasar, S. , & Gumus, O. (2016). Spatiotemporal modeling of watershed nutrient transport dynamics: Implications for eutrophication abatement. Ecological Informatics, 34, 52–69. 10.1016/j.ecoinf.2016.04.012

[jeq220551-bib-0045] Guo, Y. , Wang, X. , Zhou, L. , Melching, C. , & Li, Z. (2020). Identification of critical source areas of nitrogen load in the Miyun Reservoir watershed under different hydrological conditions. Sustainability, 12(3), 964. 10.3390/su12030964

[jeq220551-bib-0046] Hahn, C. , Prasuhn, V. , Stamm, C. , Lazzarotto, P. , Evangelou, M. W. H. , & Schulin, R. (2013). Prediction of dissolved reactive phosphorus losses from small agricultural catchments: Calibration and validation of a parsimonious model. Hydrology and Earth System Sciences, 17(10), 3679–3693. 10.5194/hess-17-3679-2013

[jeq220551-bib-0047] Hall, J. (2011). Scott River hydrological and nutrient model: Construction and calibration . MODSIM2011, 19th International Congress on Modelling and Simulation, Perth, Australia, 12–16 December 2011.

[jeq220551-bib-0048] Hall, J. , Gunarathne, G. , Herron, A. , Cresswell, R. , & Walton, M. (2018). Evaluating the effect of farm management on nutrient inflows to receiving water bodies in WA using paddock and catchment models . Hydrology and Water Resources Symposium (HWRS 2018): Water and Communities; Engineers Australia, Melbourne; pp. 314–328.

[jeq220551-bib-0049] Harmel, R. D. , Torbert, H. A. , DeLaune, P. B. , Haggard, B. E. , & Haney, R. L. (2005). Field evaluation of three phosphorus indices on new application sites in Texas. Journal of Soil and Water Conservation, 60(1), 29–42.

[jeq220551-bib-0050] Hayes, E. , Higgins, S. , Geris, J. , Nicholl, G. , & Mullan, D. (2023). Weighted risk assessment of critical source areas for soil phosphorus losses through surface runoff mechanisms. Catena, 225, 107027. 10.1016/j.catena.2023.107027

[jeq220551-bib-0051] Haygarth, P. M. , Condron, L. M. , Heathwaite, A. L. , Turner, B. L. , & Harris, G. P. (2005). The phosphorus transfer continuum: Linking source to impact with an interdisciplinary and multi‐scaled approach. Science of The Total Environment, 344(1), 5–14. 10.1016/j.scitotenv.2005.02.001 15907506

[jeq220551-bib-0052] Haygarth, P. M. , Heathwaite, A. L. , Jarvis, S. C. , & Harrod, T. R. (2000). Hydrological factors for phosphorus transfer from agricultural soils. Advances in Agronomy, 69, 153–178.

[jeq220551-bib-0053] Haygarth, P. M. , & Jarvis, S. C. (1999). Transfer of phosphorus from agricultural soils. Advances in Agronomy, 66, 195–249.

[jeq220551-bib-0054] Haygarth, P. M. , Apsimon, H. , Betson, M. , Harris, D. , Hodgkinson, R. , & Withers, P. J. A. (2009). Mitigating diffuse phosphorus transfer from agriculture according to cost and efficiency. Journal of Environmental Quality, 38(5), 2012–2022. 10.2134/jeq2008.0102 19704144

[jeq220551-bib-0055] He, Q. , Wendland, F. , & Molkenthin, F. (2019). The analysis of nitrogen load and simulation uncertainty using SWAT in a catchment with paddy field in China. Water Science and Technology, 80(4), 806–816. 10.2166/wst.2019.326 31661459

[jeq220551-bib-0056] Heathwaite, A. L. , Dils, R. M. , Liu, S. , Carvalho, L. , Brazier, R. E. , Pope, L. , Hughes, M. , Phillips, G. , & May, L. (2005). A tiered risk‐based approach for predicting diffuse and point source phosphorus losses in agricultural areas. Science of The Total Environment, 344(1), 225–239. 10.1016/j.scitotenv.2005.02.034 15907520

[jeq220551-bib-0057] Heathwaite, A. L. , Fraser, A. I. , Johnes, P. J. , Hutchins, M. , Lord, E. , & Butterfield, D. (2003). The Phosphorus Indicators Tool: A simple model of diffuse P loss from agricultural land to water. Soil Use and Management, 19(1), 1–11. 10.1111/j.1475-2743.2003.tb00273.x

[jeq220551-bib-0058] Heathwaite, A. L. , Quinn, P. F. , & Hewett, C. J. M. (2005). Modelling and managing critical source areas of diffuse pollution from agricultural land using flow connectivity simulation. Journal of Hydrology, 304(1–4), 446–461. 10.1016/j.jhydrol.2004.07.043

[jeq220551-bib-0059] Huang, N. , Lin, T. , Guan, J. , Zhang, G. , Qin, X. , Liao, J. , Liu, Q. , & Huang, Y. (2021). Identification and regulation of critical source areas of non‐point source pollution in medium and small watersheds based on source‐sink theory. Land, 10(7), 668. 10.3390/land10070668

[jeq220551-bib-0060] Hughes, K. J. , Magette, W. L. , & Kurz, I. (2005). Identifying critical source areas for phosphorus loss in Ireland using field and catchment scale ranking schemes. Journal of Hydrology, 304(1–4), 430–445. 10.1016/j.jhydrol.2004.07.042

[jeq220551-bib-0061] Iho, A. , Lankoski, J. , Ollikainen, M. , Puustinen, M. , & Lehtimäki, J. (2014). Agri‐environmental auctions for phosphorus load reduction: Experiences from a Finnish pilot. Australian Journal of Agricultural and Resource Economics, 58(2), 205–222. 10.1111/1467-8489.12049

[jeq220551-bib-0062] Iho, A. , & Laukkanen, M. (2012). Precision phosphorus management and agricultural phosphorus loading. Ecological Economics, 77, 91–102. 10.1016/j.ecolecon.2012.02.010

[jeq220551-bib-0063] Iho, A. , Valve, H. , Ekholm, P. , Uusitalo, R. , Lehtoranta, J. , Soinne, H. , & Salminen, J. (2023). Efficient protection of the Baltic Sea needs a revision of phosphorus metric. Ambio, 52(8), 1389–1399. 10.1007/s13280-023-01851-2 37036584 PMC10271980

[jeq220551-bib-0064] Imani, S. , Delavar, M. , & Niksokhan, M. H. (2019). Identification of nutrients critical source areas with SWAT model under limited data condition. Water Resources, 46(1), 128–137. 10.1134/S0097807819010147

[jeq220551-bib-0065] Keenleyside, C. , Allen, B. , Hart, K. , Menadue, H. , Stefanova, V. , Prazan, J. , Herzon, I. , Clement, T. , Povellato, A. , Maciejczak, M. , & Boatman, N. (2011). Delivering environmental benefits through entry‐level agri‐environment schemes in the EU. Institute for European Environmental Policy.

[jeq220551-bib-0066] King, K. W. , Williams, M. R. , Macrae, M. L. , Fausey, N. R. , Frankenberger, J. , Smith, D. R. , Kleinman, P. J. A. , & Brown, L. C. (2015). Phosphorus transport in agricultural subsurface drainage: A review. Journal of Environmental Quality, 44(2), 467–485. 10.2134/jeq2014.04.0163 26023966

[jeq220551-bib-0067] Kleinman, P. , Sharpley, A. , Buda, A. , McDowell, R. , & Allen, A. (2011). Soil controls of phosphorus in runoff: Management barriers and opportunities. Canadian Journal of Soil Science, 91(3), 329–338. 10.4141/cjss09106

[jeq220551-bib-0068] Kleinman, P. J. A. , Sharpley, A. N. , McDowell, R. W. , Flaten, D. N. , Buda, A. R. , Tao, L. , Bergstrom, L. , & Zhu, Q. (2011). Managing agricultural phosphorus for water quality protection: Principles for progress. Plant and Soil, 349(1–2), 169–182. 10.1007/s11104-011-0832-9

[jeq220551-bib-0069] Kleinman, P. J. A. , Smith, D. R. , Bolster, C. H. , & Easton, Z. M. (2015). Phosphorus fate, management, and modeling in artificially drained systems. Journal of Environmental Quality, 44(2), 460–466. 10.2134/jeq2015.02.0090 26023965

[jeq220551-bib-0070] Kleinman, P. J. A. , Srinivasan, M. S. , Dell, C. J. , Schmidt, J. P. , Sharpley, A. N. , & Bryant, R. B. (2006). Role of rainfall intensity and hydrology in nutrient transport via surface runoff. Journal of Environmental Quality, 35(4), 1248–1259. 10.2134/jeq2006.0015 16825444

[jeq220551-bib-0071] Kovacs, A. S. , Fulop, B. , & Honti, M. (2012). Detection of hot spots of soil erosion and reservoir siltation in ungauged Mediterranean catchments. Energy Procedia, 18, 934–943.

[jeq220551-bib-0072] Ladson, T. , & Tilleard, J. (2006). BMPs for reducing phosphorus loads to the Gippsland Lakes . Gippsland Lakes Task Force.

[jeq220551-bib-0073] Lane, S. N. , Brookes, C. J. , Louise Heathwaite, A. , & Reaney, S. (2006). Surveillant science: Challenges for the management of rural environments emerging from the new generation diffuse pollution models. Journal of Agricultural Economics, 57(2), 239–257. 10.1111/j.1477-9552.2006.00050.x

[jeq220551-bib-0074] Lankoski, J. (2006). Alternative approaches for evaluating the performance of buffer strip policy in Finland . OECD workshop on evaluating agri‐environmental policies.

[jeq220551-bib-0075] Lemunyon, J. L. , & Gilbert, R. G. (1993). The concept and need for a phosphorus assessment tool. Journal of Production Agriculture, 6(4), 483–486. 10.2134/jpa1993.0483

[jeq220551-bib-0076] Li, S. , Zhang, L. , Du, Y. , Liu, H. , Zhuang, Y. , & Liu, S. (2016). Evaluating phosphorus loss for watershed management: Integrating a weighting scheme of watershed heterogeneity into export coefficient model. Environmental Modeling and Assessment, 21(5), 657–668. 10.1007/s10666-016-9499-1

[jeq220551-bib-0077] Li, H. , Zhou, X. , Huang, K. , Hao, G. , & Li, J. (2022). Research on optimal control of non‐point source pollution: A case study from the Danjiang River basin in China. Environmental Science and Pollution Research, 29(11), 15582–15602. 10.1007/s11356-021-16740-y 34628618

[jeq220551-bib-0078] Lilburne, L. , Carrick, S. , Webb, T. , & Moir, J. (2012). Computer‐based evaluation of methods to sample nitrate leached from grazed pasture. Soil Use and Management, 28(1), 19–26. 10.1111/j.1475-2743.2011.00378.x

[jeq220551-bib-0079] Liu, J. , Kleinman, P. J. A. , Aronsson, H. , Flaten, D. , McDowell, R. W. , Bechmann, M. , Beegle, D. B. , Robinson, T. P. , Bryant, R. B. , Liu, H. , Sharpley, A. N. , & Veith, T. L. (2018). A review of regulations and guidelines related to winter manure application. Ambio, 47, 657–670. 10.1007/s13280-018-1012-4 29397547 PMC6131135

[jeq220551-bib-0080] Lou, H. , Yang, S. , Zhao, C. , Shi, L. , Wu, L. , Wang, Y. , & Wang, Z. (2016). Detecting and analyzing soil phosphorus loss associated with critical source areas using a remote sensing approach. Science of the Total Environment, 573, 397–408. 10.1016/j.scitotenv.2016.08.048 27572533

[jeq220551-bib-0081] Lu, J. , Garzon‐Garcia, A. , Hamilton, D. P. , Burton, J. , & Burford, M. A. (2023). A slurry approach to identify nutrient critical source areas from subtropical catchment erosion. Journal of Environmental Management, 343, 118187. 10.1016/j.jenvman.2023.118187 37235987

[jeq220551-bib-0082] Lucci, G. M. , McDowell, R. W. , & Condron, L. M. (2013). Phosphorus source areas in a dairy catchment in Otago, New Zealand. Soil Research, 50(2), 145–156. 10.1071/sr12030

[jeq220551-bib-0083] Maas, R. P. , Smolen, M. D. , & Dressing, S. A. (1985). Selecting critical areas for nonpoint‐source pollution control. Journal of Soil and Water Conservation, 40(1), 68–71.

[jeq220551-bib-0084] Margenot, A. J. , Zhou, S. , McDowell, R. , Hebert, T. , Fox, G. , Schilling, K. , Richmond, S. , Kovar, J. L. , Wickramarathne, N. , Lemke, D. , Boomer, K. , & Golovay, S. (2023). Streambank erosion and phosphorus loading to surface waters: Knowns, unknowns, and implications for nutrient loss reduction research and policy. Journal of Environmental Quality, 52(6), 1063–1079. 10.1002/jeq2.20514 37725393

[jeq220551-bib-0085] McCloskey, G. L. , Baheerathan, R. , Dougall, C. , Ellis, R. , Bennett, F. R. , Waters, D. , Darr, S. , Fentie, B. , Hateley, L. R. , & Askildsen, M. (2021). Modelled estimates of dissolved inorganic nitrogen exported to the Great Barrier Reef lagoon. Marine Pollution Bulletin, 171, 112655. 10.1016/j.marpolbul.2021.112655 34265552

[jeq220551-bib-0086] McDowell, R. W. (2006). Phosphorus and sediment loss in a catchment with winter forage grazing of cropland by dairy cattle. Journal of Environmental Quality, 35(2), 575–583. 10.2134/jeq2005.0364 16510702

[jeq220551-bib-0087] McDowell, R. W. (2014). Estimating the mitigation of anthropogenic loss of phosphorus in New Zealand grassland catchments. The Science of the Total Environment, 468–469, 1178–1186. 10.1016/j.scitotenv.2013.03.056 23579204

[jeq220551-bib-0088] McDowell, R. W. (2017). Does variable rate irrigation decrease nutrient leaching losses from grazed dairy farming? Soil Use and Management, 33(4), 530–537. 10.1111/sum.12363

[jeq220551-bib-0089] McDowell, R. W. , Daly, K. , & Fenton, O. (2020). Mitigation of phosphorus, sediment and *Escherichia coli* losses in runoff from a dairy farm roadway. Irish Journal of Agricultural and Food Research, 59(1), 201–205. 10.15212/ijafr-2020-0117

[jeq220551-bib-0090] McDowell, R. W. , Depree, C. , & Stenger, R. (2020). Likely controls on dissolved reactive phosphorus concentrations in baseflow of an agricultural stream. Journal of Soils and Sediments, 20(8), 3254–3265. 10.1007/s11368-020-02644-w

[jeq220551-bib-0091] McDowell, R. W. , Drewry, J. J. , Muirhead, R. W. , & Paton, R. J. (2003). Cattle treading and phosphorus and sediment loss in overland flow from grazed cropland. Soil Research, 41(8), 1521–1532. 10.1071/SR03042

[jeq220551-bib-0092] McDowell, R. W. , Hedley, M. J. , Pletnyakov, P. , Rissmann, C. , Catto, W. , & Patrick, W. (2019). Why are median phosphorus concentrations improving in New Zealand streams and rivers? Journal of the Royal Society of New Zealand, 49(2), 143–170. 10.1080/03036758.2019.1576213

[jeq220551-bib-0093] McDowell, R. W. , Lucci, G. M. , Peyroux, G. , Yoswara, H. , Brown, M. , Kalmakoff, I. , Cox, N. R. , Smale, P. , Wheeler, D. M. , Watkins, N. , Smith, C. W. , Monaghan, R. M. , Muirhead, R. W. , Catto, W. , & Risk, J. (2015). An assessment of MitAgator: A farm‐scale tool to estimate and manage the loss of contaminants from land to water. Transactions of the ASABE, 59(2), 537–543. 10.13031/trans.59.11192

[jeq220551-bib-0094] McDowell, R. W. , Macintosh, K. A. , & Depree, C. (2023). Linking the uptake of best management practices on dairy farms to catchment water quality improvement over a 20‐year period. Science of The Total Environment, 895, 164963. 10.1016/j.scitotenv.2023.164963 37348722

[jeq220551-bib-0095] McDowell, R. W. , & Monaghan, R. M. (2015). Extreme phosphorus losses in drainage from grazed dairy pastures on marginal land. Journal of Environmental Quality, 44(2), 545–551. 10.2134/jeq2014.04.0160 26023973

[jeq220551-bib-0096] McDowell, R. W. , Monaghan, R. M. , & Morton, J. (2003). Soil phosphorus concentrations to minimise potential P loss to surface waters in Southland. New Zealand Journal of Agricultural Research, 46(3), 239–253. 10.1080/00288233.2003.9513550

[jeq220551-bib-0097] McDowell, R. W. , Monaghan, R. M. , & Wheeler, D. (2005). Modelling phosphorus losses from pastoral farming systems in New Zealand. New Zealand Journal of Agricultural Research, 48(1), 131–141. 10.1080/00288233.2005.9513643

[jeq220551-bib-0098] McDowell, R. W. , & Nash, D. (2012). A review of the cost‐effectiveness and suitability of mitigation strategies to prevent phosphorus loss from dairy farms in New Zealand and Australia. Journal of Environmental Quality, 41(3), 680–693. 10.2134/jeq2011.0041 22565250

[jeq220551-bib-0099] McDowell, R. W. , Noble, A. , Pletnyakov, P. , Haggard, B. E. , & Mosley, L. M. (2020). Global mapping of freshwater nutrient enrichment and periphyton growth potential. Scientific Reports, 10(1), 3568. 10.1038/s41598-020-60279-w 32107412 PMC7046692

[jeq220551-bib-0100] McDowell, R. W. , Pletnyakov, P. , Lim, A. , & Salmon, G. (2021). Implications of water quality policy on land use: A case study of the approach in New Zealand. Marine and Freshwater Research, 72(4), 451–455. 10.1071/MF20201

[jeq220551-bib-0101] McDowell, R. W. , & Sharpley, A. N. (2001). Approximating phosphorus release from soils to surface runoff and subsurface drainage. Journal of Environmental Quality, 30, 508–520. 11285912 10.2134/jeq2001.302508x

[jeq220551-bib-0102] McDowell, R. W. , Sharpley, A. N. , Beegle, D. B. , & Weld, J. L. (2001). Comparing phosphorus management strategies at a watershed scale. Journal of Soil and Water Conservation, 56(4), 306–315.

[jeq220551-bib-0103] McDowell, R. W. , & Srinivasan, M. S. (2009). Identifying critical source areas for water quality: 2. Validating the approach for phosphorus and sediment losses in grazed headwater catchments. Journal of Hydrology, 379, 68–80. 10.1016/j.jhydrol.2009.09.045

[jeq220551-bib-0104] Ministry for the Environment . (2022). National Policy Statement for Freshwater Management 2020 . Author.

[jeq220551-bib-0105] Mogollón, J. M. , Bouwman, A. F. , Beusen, A. H. W. , Lassaletta, L. , Van Grinsven, H. J. M. , & Westhoek, H. (2021). More efficient phosphorus use can avoid cropland expansion. Nature Food, 2(7), 509–518. 10.1038/s43016-021-00303-y 37117673

[jeq220551-bib-0106] Monaghan, R. M. , & Smith, L. C. (2004). Minimising surface water pollution resulting from farm‐dairy effluent application to mole‐pipe drained soils. II. The contribution of preferential flow of effluent to whole‐farm pollutant losses in subsurface drainage from a West Otago dairy farm. New Zealand Journal of Agricultural Research, 47(4), 417–428. 10.1080/00288233.2004.9513610

[jeq220551-bib-0107] Monaghan, R. M. , & Smith, L. C. (2012). Contaminant losses in overland flow from dairy farm laneways in southern New Zealand. Agriculture, Ecosystems & Environment, 159, 170–175. 10.1016/j.agee.2012.07.022

[jeq220551-bib-0108] Murphy, P. N. C. , Mellander, P. E. , Melland, A. R. , Buckley, C. , Shore, M. , Shortle, G. , Wall, D. P. , Treacy, M. , Shine, O. , Mechan, S. , & Jordan, P. (2015). Variable response to phosphorus mitigation measures across the nutrient transfer continuum in a dairy grassland catchment. Agriculture, Ecosystems & Environment, 207, 192–202. 10.1016/j.agee.2015.04.008

[jeq220551-bib-0109] Nagireddy, N. R. , Keesara, V. R. , Sridhar, V. , & Srinivasan, R. (2022). Streamflow and sediment yield analysis of two medium‐sized east‐flowing river basins of India. Water, 14(19), 2960. 10.3390/w14192960

[jeq220551-bib-0110] Nash, D. M. , McDowell, R. W. , Condron, L. M. , & Mclaughlin, M. J. (2019). Direct exports of phosphorus from fertilizers applied to grazed pastures. Journal of Environmental Quality, 48(5), 1380–1396. 10.2134/jeq2019.02.0085 31589740

[jeq220551-bib-0111] Nelson, N. O. , & Shober, A. L. (2012). Evaluation of phosphorus indices after twenty years of science and development. Journal of Environmental Quality, 41(6), 1703–1710. 10.2134/jeq2012.0342 23128727

[jeq220551-bib-0112] Niraula, R. , Kalin, L. , Srivastava, P. , & Anderson, C. J. (2013). Identifying critical source areas of nonpoint source pollution with SWAT and GWLF. Ecological Modelling, 268, 123–133. 10.1016/j.ecolmodel.2013.08.007

[jeq220551-bib-0113] Ockenden, M. C. , Deasy, C. E. , Benskin, C. M. H. , Beven, K. J. , Burke, S. , Collins, A. L. , Evans, R. , Falloon, P. D. , Forber, K. J. , Hiscock, K. M. , Hollaway, M. J. , Kahana, R. , Macleod, C. J. A. , Reaney, S. M. , Snell, M. A. , Villamizar, M. L. , Wearing, C. , Withers, P. J. A. , Zhou, J. G. , & Haygarth, P. M. (2016). Changing climate and nutrient transfers: Evidence from high temporal resolution concentration‐flow dynamics in headwater catchments. Science of the Total Environment, 548–549, 325–339. 10.1016/j.scitotenv.2015.12.086 26803731

[jeq220551-bib-0114] Ockenden, M. C. , Hollaway, M. J. , Beven, K. J. , Collins, A. L. , Evans, R. , Falloon, P. D. , Forber, K. J. , Hiscock, K. M. , Kahana, R. , Macleod, C. J. A. , Tych, W. , Villamizar, M. L. , Wearing, C. , Withers, P. J. A. , Zhou, J. G. , Barker, P. A. , Burke, S. , Freer, J. E. , Johnes, P. J. , … Haygarth, P. M. (2017). Major agricultural changes required to mitigate phosphorus losses under climate change. Nature Communications, 8(1), 161. 10.1038/s41467-017-00232-0 PMC5534432 28757602

[jeq220551-bib-0115] Oliver, D. M. , Page, T. , Hodgson, C. J. , Heathwaite, A. L. , Chadwick, D. R. , Fish, R. D. , & Winter, M. (2010). Development and testing of a risk indexing framework to determine field‐scale critical source areas of faecal bacteria on grassland. Environmental Modelling and Software, 25(4), 503–512. 10.1016/j.envsoft.2009.10.003

[jeq220551-bib-0116] Osmond, D. L. , Kleinman, P. J. A. , Coale, F. , Nelson, N. , Bolster, C. , & McGrath, J. (2023). A short history of the phosphorus index and Andrew Sharpley's contributions from inception through development and implementation. Journal of Environmental Quality. 10.1002/jeq2.20535 PMC12265852 38128917

[jeq220551-bib-0117] Osmond, D. L. , Meals, D. W. , Arabi, M. , & Hoag, D. L. (2012). Cannonsville Reservoir, New York: National Institute of Food and Agriculture–Conservation Effects Assessment Project. In D. L. Osmond , D. W. Meals , D. L. K. Hoag , & M. Arabi (Eds.), How to build better agricultural conservation programs to protect water quality (pp. 296–315). Soil and Water Conservation Society.

[jeq220551-bib-0118] Osmond, D. , Sharpley, A. , Bolster, C. , Cabrera, M. , Feagley, S. , Lee, B. , Mitchell, C. , Mylavarapu, R. , Oldham, L. , Walker, F. , & Zhang, H. (2012). Comparing phosphorus indices from twelve southern U.S. states against monitored phosphorus loads from six prior southern studies. Journal of Environmental Quality, 41(6), 1741–1749. 10.2134/jeq2012.0013 23128731

[jeq220551-bib-0119] Page, T. , Haygarth, P. M. , Beven, K. J. , Joynes, A. , Butler, T. , Keeler, C. , Freer, J. , Owens, P. N. , & Wood, G. A. (2005). Spatial variability of soil phosphorus in relation to the topographic index and critical source areas: Sampling for assessing risk to water quality. Journal of Environmental Quality, 34(6), 2263–2277. 10.2134/jeq2004.0398 16275728

[jeq220551-bib-0120] Pareto, V. (1897). Cours d’économie politique (Vol. 2). Rouge.

[jeq220551-bib-0121] Perez, M. R. (2015). Regulating farmer nutrient management: A three‐state case study on the Delmarva Peninsula. Journal of Environmental Quality, 44(2), 402–414. 10.2134/jeq2014.07.0304 26023959

[jeq220551-bib-0122] Pionke, H. B. , Gburek, W. J. , & Sharpley, A. N. (2000). Critical source area controls on water quality in an agricultural watershed located in the Chesapeake Basin. Ecological Engineering, 14(4), 325–335. 10.1016/S0925-8574(99)00059-2

[jeq220551-bib-0123] Preedy, N. , Mctiernan, K. , Matthews, R. , Heathwaite, L. , & Haygarth, P. (2001). Rapid incidental phosphorus transfers from grassland. Journal of Environmental Quality, 30, 2105–2112. 11790020 10.2134/jeq2001.2105

[jeq220551-bib-0124] Reaney, S. M. , Mackay, E. B. , Haygarth, P. M. , Fisher, M. , Molineux, A. , Potts, M. , & Benskin, C. M. H. (2019). Identifying critical source areas using multiple methods for effective diffuse pollution mitigation. Journal of Environmental Management, 250, 109366. 10.1016/j.jenvman.2019.109366 31494409

[jeq220551-bib-0125] Keith Reid, D. (2011). A modified Ontario P index as a tool for on‐farm phosphorus management. Canadian Journal of Soil Science, 91(3), 455–466. 10.4141/cjss09088

[jeq220551-bib-0126] Remund, D. , Liebisch, F. , Liniger, H. P. , Heinimann, A. , & Prasuhn, V. (2021). The origin of sediment and particulate phosphorus inputs into water bodies in the Swiss Midlands – A twenty‐year field study of soil erosion. Catena, 203, 105290. 10.1016/j.catena.2021.105290

[jeq220551-bib-0127] Robinson, J. S. , Sharpley, A. N. , & Smith, S. J. (1994). Development of a method to determine bioavailable phosphorus loss in agricultural runoff. Agriculture, Ecosystems & Environment, 47(4), 287–297. 10.1016/0167-8809(94)90095-7

[jeq220551-bib-0128] Scholefield, P. , Heathwaite, A. L. , Brazier, R. E. , Page, T. , Schärer, M. , Beven, K. , Hodgkinson, R. , Withers, P. , Walling, D. , & Haygarth, P. M. (2013). Estimating phosphorus delivery from land to water in headwater catchments using a fuzzy decision tree approach. Soil Use and Management, 29(s1), 175–186. 10.1111/sum.12007

[jeq220551-bib-0129] Schoumans, O. F. (2015). Phosphorus leaching from soils: Process description, risk assessment and mitigation . Alterra Wageningen.

[jeq220551-bib-0130] Schoumans, O. F. , & Chardon, W. J. (2003). Risk assessment methodologies for predicting phosphorus losses. Journal of Plant Nutrition and Soil Science, 166(4), 403–408. 10.1002/jpln.200321222

[jeq220551-bib-0131] Schoumans, O. F. , & Chardon, W. J. (2015). Phosphate saturation degree and accumulation of phosphate in various soil types in the Netherlands. Geoderma, 237–238, 325–335. 10.1016/j.geoderma.2014.08.015

[jeq220551-bib-0132] Schoumans, O. F. , Van Der Salm, C. , & Groenendijk, P. (2013). PLEASE: A simple model to determine P losses by leaching. Soil Use and Management, 29(s1), 138–146. 10.1111/sum.12008

[jeq220551-bib-0133] Shang, X. , Wang, X. , Zhang, D. , Chen, W. , Chen, X. , & Kong, H. (2012). An improved SWAT‐based computational framework for identifying critical source areas for agricultural pollution at the lake basin scale. Ecological Modelling, 226, 1–10. 10.1016/j.ecolmodel.2011.11.030

[jeq220551-bib-0134] Sharpley, A. N. (1980). The effect of storm interval on the transport of soluble phosphorus in runoff. Journal of Environmental Quality, 9(4), 575–578.

[jeq220551-bib-0135] Sharpley, A. N. (1985). Depth of surface soil‐runoff interaction as affected by rainfall, soil slope, and management. Soil Science Society of America Journal, 49(4), 1010–1015.

[jeq220551-bib-0136] Sharpley, A. N. (1987). Relative availabilities of native, residual, and fertilizer phosphorus to winter wheat. Soil Science Society of America Journal, 51(6), 1531–1535.

[jeq220551-bib-0137] Sharpley, A. , Beegle, D. , Bolster, C. , Good, L. , Joern, B. , Ketterings, Q. , Lory, J. , Mikkelsen, R. , Osmond, D. , & Vadas, P. (2011). Revision of the 590 nutrient management standard: SERA‐17 recommendations . Southern Cooperative Series Bulletin No. 412.

[jeq220551-bib-0138] Sharpley, A. , Jarvie, H. P. , Buda, A. , May, L. , Spears, B. , & Kleinman, P. (2013). Phosphorus legacy: Overcoming the effects of past management practices to mitigate future water quality impairment. Journal of Environmental Quality, 42(5), 1308–1326. 10.2134/jeq2013.03.0098 24216410

[jeq220551-bib-0139] Sharpley, A. , Kleinman, P. , Baffaut, C. , Beegle, D. , Bolster, C. , Collick, A. , Easton, Z. , Lory, J. , Nelson, N. , Osmond, D. , Radcliffe, D. , Veith, T. , & Weld, J. (2017). Evaluation of phosphorus site assessment tools: Lessons from the USA. Journal of Environmental Quality, 46(6), 1250–1256. 10.2134/jeq2016.11.0427 29293829

[jeq220551-bib-0140] Sharpley, A. N. , Kleinman, P. J. A. , Heathwaite, A. L. , Gburek, W. J. , Folmar, G. J. , & Schmidt, J. P. (2008). Phosphorus loss from an agricultural watershed as a function of storm size. Journal of Environmental Quality, 37(2), 362–368. 10.2134/jeq2007.0366 18268298

[jeq220551-bib-0141] Sharpley, A. N. , McDowell, R. W. , Weld, J. L. , & Kleinman, P. J. A. (2001). Assessing site vulnerability to phosphorus loss in an agricultural watershed. Journal of Environmental Quality, 30(6), 2026–2036. 10.2134/jeq2001.2026 11790010

[jeq220551-bib-0142] Sharpley, A. , Richards, P. , Herron, S. , & Baker, D. (2012). Case study comparison between litigated and voluntary nutrient management strategies. Journal of Soil and Water Conservation, 67(5), 442–450. 10.2489/jswc.67.5.442

[jeq220551-bib-0143] Sharpley, A. N. , & Smith, S. J. (1989). Prediction of soluble phosphorus transport in agricultural runoff. Journal of Environmental Quality, 18(3), 313–316.

[jeq220551-bib-0144] Sharpley, A. N. , Smith, S. J. , & Menzel, R. G. (1982). Prediction of phosphorus losses in runoff from Southern Plains watersheds. Journal of Environmental Quality, 11(2), 247–251.

[jeq220551-bib-0145] Sharpley, A. N. , & Syers, J. K. (1979). Phosphorus inputs into a stream draining an agricultural watershed. II: Amounts contributed and relative significance of runoff types. Water, Air, & Soil Pollution, 11(4), 417–428. 10.1007/bf00283433

[jeq220551-bib-0146] Sharpley, A. N. , Weld, J. L. , Beegle, D. B. , Kleinman, P. J. A. , Gburek, W. J. , Moore, P. A. , & Mullins, G. (2003). Development of phosphorus indices for nutrient management planning strategies in the United States. Journal of Soil and Water Conservation, 58(3), 137–152.

[jeq220551-bib-0147] Shober, A. L. , Buda, A. R. , Turner, K. C. , Fiorellino, N. M. , Andres, A. S. , Mcgrath, J. M. , & Sims, J. T. (2017). Assessing coastal plain risk indices for subsurface phosphorus loss. Journal of Environmental Quality, 46(6), 1270–1286. 10.2134/jeq2017.03.0102 29293841

[jeq220551-bib-0148] Shrestha, N. K. , Rudra, R. P. , Daggupati, P. , Goel, P. K. , & Shukla, R. (2021). A comparative evaluation of the continuous and event‐based modelling approaches for identifying critical source areas for sediment and phosphorus losses. Journal of Environmental Management, 277. 10.1016/j.jenvman.2020.111427 33069154

[jeq220551-bib-0149] Spiegal, S. , Kleinman, P. J. A. , Endale, D. M. , Bryant, R. B. , Dell, C. , Goslee, S. , Meinen, R. J. , Flynn, K. C. , Baker, J. M. , Browning, D. M. , Mccarty, G. , Bittman, S. , Carter, J. , Cavigelli, M. , Duncan, E. , Gowda, P. , Li, X. , Ponce‐Campos, G. E. , Cibin, R. , … Yang, Q. (2020). Manuresheds: Advancing nutrient recycling in US agriculture. Agricultural Systems, 182, 102813. 10.1016/j.agsy.2020.102813

[jeq220551-bib-0150] Strauss, P. , Leone, A. , Ripa, M. N. , Turpin, N. , Lescot, J.‐M. , & Laplana, R. (2007). Using critical source areas for targeting cost‐effective best management practices to mitigate phosphorus and sediment transfer at the watershed scale. Soil Use and Management, 23(1), 144–153. 10.1111/j.1475-2743.2007.00118.x

[jeq220551-bib-0151] Thomas, I. A. , Mellander, P.‐E. , Murphy, P. N. C. , Fenton, O. , Shine, O. , Djodjic, F. , Dunlop, P. , & Jordan, P. (2016). A sub‐field scale critical source area index for legacy phosphorus management using high resolution data. Agriculture, Ecosystems and Environment, 233, 238–252. 10.1016/j.agee.2016.09.012

[jeq220551-bib-0152] Trevisan, D. , Dorioz, J. M. , Poulenard, J. , Quetin, P. , Prigent Combaret, C. , & Merot, P. (2010). Mapping of critical source areas for diffuse fecal bacterial pollution in extensively grazed watersheds. Water Research, 44(13), 3847–3860. 10.1016/j.watres.2010.04.039 20569961

[jeq220551-bib-0153] Ulén, B. , Djodjic, F. , Etana, A. , Johansson, G. , & Lindström, J. (2011). The need for an improved risk index for phosphorus losses to water from tile‐drained agricultural land. Journal of Hydrology, 400(1), 234–243. 10.1016/j.jhydrol.2011.01.038

[jeq220551-bib-0154] Ulén, B. , Johansson, G. , & Kyllmar, K. (2001). Model predictions and long‐term trends in phosphorus transport from arable lands in Sweden. Agricultural Water Management, 49(3), 197–210. 10.1016/S0378-3774(00)00145-1

[jeq220551-bib-0155] United States Department of Agriculture (USDA), & United States Environmental Protection Agency (USEPA) . (1999). Unified National Strategy for Animal Feeding Operations. Author.

[jeq220551-bib-0156] Uusi‐Kämppä, J. , & Jauhiainen, L. (2010). Long‐term monitoring of buffer zone efficiency under different cultivation techniques in boreal conditions. Agriculture, Ecosystems & Environment, 137(1), 75–85. 10.1016/j.agee.2010.01.002

[jeq220551-bib-0157] Uusitalo, R. , Turtola, E. , & Grönroos, J. (2007). Finnish trends in phosphorus balances and soil test phosphorus. Agricultural and Food Science, 16(4), 301–316. 10.2137/145960607784125339

[jeq220551-bib-0158] Veith, T. L. , Sharpley, A. N. , Weld, J. L. , & Gburek, W. J. (2005). Comparison of measured and simulated phosphorus losses with indexed site vulnerability. Transactions of the American Society of Agricultural Engineers, 48, 557–565.

[jeq220551-bib-0159] Verhoeven, T. J. (1992). Blue‐green algae: Final report of the New South Wales Blue‐Green Algae Task Force . Department of Water Resources for the Task Force.

[jeq220551-bib-0160] Victorian Environment Protection Authority . (1995). State environment protection policy (Waters of Victoria) schedule F5 ‐ The Latrobe and Thompson River Basins and Merriman Creek catchment . Author.

[jeq220551-bib-0161] Victorian Environment Protection Authority . (2018). Environment Protection Act . Author.

[jeq220551-bib-0162] Wagena, M. B. , Collick, A. S. , Ross, A. C. , Najjar, R. G. , Rau, B. , Sommerlot, A. R. , Fuka, D. R. , Kleinman, P. J. A. , & Easton, Z. M. (2018). Impact of climate change and climate anomalies on hydrologic and biogeochemical processes in an agricultural catchment of the Chesapeake Bay watershed, USA. Science of The Total Environment, 637–638, 1443–1454. 10.1016/j.scitotenv.2018.05.116 29801237

[jeq220551-bib-0163] Wall, D. , Jordan, P. , Melland, A. R. , Mellander, P.‐E. , Buckley, C. , Reaney, S. M. , & Shortle, G. (2011). Using the nutrient transfer continuum concept to evaluate the European Union Nitrates Directive National Action Programme. Environmental Science & Policy, 14(6), 664–674. 10.1016/j.envsci.2011.05.003

[jeq220551-bib-0164] Warren, R. (2023). Developers can expect ‘nutrient neutrality’ debate to rumble through autumn . Pinsent Masons. https://www.pinsentmasons.com/out‐law/analysis/developers‐expect‐nutrient‐neutrality‐debate‐rumble‐through‐autumn

[jeq220551-bib-0165] Waters, S. , & Webster‐Brown, J. G. (2016). The use of a mass balance phosphorus budget for informing nutrient management in shallow coastal lakes. Journal of Hydro‐Environment Research, 10, 32–49. 10.1016/j.jher.2015.11.002

[jeq220551-bib-0166] Wei, P. , Ouyang, W. , Gao, X. , Hao, F. , Hao, Z. , & Liu, H. (2017). Modified control strategies for critical source area of nitrogen (CSAN) in a typical freeze‐thaw watershed. Journal of Hydrology, 551, 518–531. 10.1016/j.jhydrol.2017.06.026

[jeq220551-bib-0167] Wen, W. , Zhuang, Y. , Zhang, L. , Li, S. , Ruan, S. , & Zhang, Q. (2021). Preferred hierarchical control strategy of phosphorus from non‐point source pollution at regional scale. Environmental Science and Pollution Research, 28(42), 60111–60121. 10.1007/s11356-021-14138-4 34155589

[jeq220551-bib-0168] West Gippsland Catchment Management Authority (WGCMA) . (2018). Lake Wellington Lake and Water Management Plan ‐ A plan for sustainable irrigation in the Lake Wellington Catchment 2018–2028 . Author.

[jeq220551-bib-0169] White, M. D. , Metherell, A. K. , & Roberts, A. H. C. (2017). The use of variable rate fertiliser applications in NZ hill country. In L. D. Currie & M. J. Hedley (Eds.), Science and policy: Nutrient management challenges for the next generation (pp. 1–13). Fertilizer and Lime Research Centre, Massey University.

[jeq220551-bib-0170] White, M. J. , Storm, D. E. , Busteed, P. R. , Stoodley, S. H. , & Phillips, S. J. (2009). Evaluating nonpoint source critical source area contributions at the watershed scale. Journal of Environmental Quality, 38(4), 1654–1663. 10.2134/jeq2008.0375 19549942

[jeq220551-bib-0171] Winchell, M. F. , Folle, S. , Meals, D. , Moore, J. , Srinivasan, R. , & Howe, E. A. (2015). Using SWAT for sub‐field identification of phosphorus critical source areas in a saturation excess runoff region. Hydrological Sciences Journal, 60(5), 844–862. 10.1080/02626667.2014.980262

[jeq220551-bib-0172] Wu, L. , Liu, X. , Chen, J. , Li, J. , Yu, Y. , & Ma, X. (2022). Efficiency assessment of best management practices in sediment reduction by investigating cost‐effective tradeoffs. Agricultural Water Management, 265, 107546. 10.1016/j.agwat.2022.107546

[jeq220551-bib-0173] Van Der Zee, S. E. A. T. M. , & Van Riemsdijk, W. H. (1988). Model for long‐term phosphate reaction kinetics in soil. Journal of Environmental Quality, 17(1), 35–41.

[jeq220551-bib-0174] Zhang, T. , Page, T. , Heathwaite, L. , Beven, K. , Oliver, D. M. , & Haygarth, P. M. (2013). Estimating phosphorus delivery with its mitigation measures from soil to stream using fuzzy rules. Soil Use and Management, 29(s1), 187–198. 10.1111/j.1475-2743.2012.00433.x

[jeq220551-bib-0175] Zhuang, Y. , Zhang, L. , Du, Y. , Yang, W. , Wang, L. , & Cai, X. (2016). Identification of critical source areas for nonpoint source pollution in the Danjiangkou Reservoir Basin, China. Lake and Reservoir Management, 32(4), 341–352. 10.1080/10402381.2016.1204396

